# 1-Pyrene Carboxylic Acid: An Internalization Enhancer for Short Oligoarginines

**DOI:** 10.3390/ijms26052202

**Published:** 2025-02-28

**Authors:** Csaba Bató, Ildikó Szabó, Mo’ath Yousef, Dorina Lenzinger, Fülöp Károly Grébecz, Tamás Visnovitz, Szilvia E. Bősze, Zoltán Bánóczi, Gábor Mező

**Affiliations:** 1 Department of Organic Chemistry, Institute of Chemistry, Faculty of Science, ELTE Eötvös Loránd University, Pázmány Péter Sétány 1/A, 1117 Budapest, Hungary; bs.csabi@gmail.com; 2 Hevesy György PhD School of Chemistry, Institute of Chemistry, ELTE Eötvös Loránd University, Pázmány Péter Sétány 1/A, 1117 Budapest, Hungary; 3 HUN-REN-ELTE Research Group of Peptide Chemistry, 1117 Budapest, Hungary; ildiko.szabo@ttk.elte.hu (I.S.); or bosze.szilvia@semmelweis.hu (S.E.B.); 4 MTA-TTK Lendület “Momentum” Peptide-Based Vaccines Research Group, Institute of Materials and Environmental Chemistry, HUN-REN Research Centre for Natural Sciences, 1117 Budapest, Hungary; 5 Ecole Super Biotechnol Strasbourg ESBS, CNRS, UMR 7242, Biotechnol & Cellular Signalling, University Strasbourg, F-67400 Illkirch Graffenstaden, France; yousefmoath@gmail.com; 6 Department of Genetics, Cell- and Immunobiology, Faculty of Medicine, Semmelweis University, Nagyvárad tér 4, 1089 Budapest, Hungary; lenzinger.dorina@semmelweis.hu (D.L.); grebecz.fulop.karoly@semmelweis.hu (F.K.G.);; 7 Department of Plant Physiology and Molecular Plant Biology, ELTE Eötvös Loránd University, Pázmány Péter Sétány 1/c, 1117 Budapest, Hungary

**Keywords:** cell-penetrating peptide, aromatic modification, transport molecule, hydrophobic molecule

## Abstract

Getting through the cell membrane is challenging, and transporting a therapeutic agent while entering the cell is even more complicated. Cell-penetrating peptides (CPPs) are valuable tools for solving this problem, although they have drawbacks. In this work, the synthesis and investigation of efficient CPPs are described. We used an aromatic group, 1-pyrene carboxylic acid (PCA), to enhance internalization. We designed oligoarginines to investigate the effect of PCA in different positions at the *N*-terminus or in the side chain. Our novel peptide derivatives showed remarkable internalization on tumor cell lines, and more than one endocytic pathway plays a role in their internalization mechanism. With this modification, there is an opportunity to design short oligoarginines that can rival well-known CPPs like octaarginine in internalization.

## 1. Introduction

Several diseases have intracellular target molecules that present the challenge of delivering therapeutic agents into the cells. This problem has grown in recent years, and one solution might be the application of cell-penetrating peptides (CCPs). These molecules may transport drugs [1,2,3,4], proteins [5,6,7,8], and other therapeutic peptides [9,10,11] into the cells.

The first CPP was discovered in 1988 when scientists examined the human immunodeficiency virus HIV-1 Tat protein [12]. In parallel, another promising CPP was derived from the Drosophila Antennapedia protein and called penetratin [13]. These peptides induced intensive research of new CPPs that may serve as drug delivery units. Both showed remarkable internalization with a common feature in their sequence: the number of arginine residues. These residues and lysines result in positively charged peptides in both cases under physiological conditions. When different positively charged oligopeptides were compared, it turned out that it was not only the positive charge, but also the arginine’s side chain functional group (guanidine group) was essential for internalization [14]. This positively charged functional group can interact with the negative cell membrane [15,16]. Based on these results, oligoarginines such as octa- [17], nona- [18,19,20], and decaarginine [21,22] are commonly used as cell-penetrating peptides.

In CPP chemistry, new members are often discovered by modifying well-known CPPs to enhance their internalization ability [17]. One of these modifications increased aromaticity by incorporating tryptophan(s) [23,24]. Initially, the important role of tryptophan was discovered when researchers substituted Trp^48^ and Trp^56^ in the sequence of penetration (RQIKIW^48^FQNRRMKW^56^KK) with phenylalanine. The cellular uptake of these derivatives decreased drastically [18]. The effect of Trp on the cellular uptake was dependent on the position of tryptophan [21]. It is favorable if a sizeable, large hydrophobic peptide surface helps interactions with the cell membrane occur.

Another way to enhance cellular uptake is the incorporation of different *N*-terminal modifications [25,26,27]. In this way, the hydrophobic peptide face can be easily facilitated. We and others described earlier that the Dabcyl group (4-((4-(dimethylamino)phenyl)azo)benzoic acid), a commonly used quencher in FRET pairs [28,29], is an excellent enhancer [30]. It can drastically increase the internalization of oligoarginines, like hexaarginine or tetraarginine [24]. While the former showed higher internalization compared to octaarginine, the internalization of the latter is far from the efficiency of octaarginine. However, combining Dabcyl and Trp residue in oligoarginines resulted in a very efficient short cell-penetrating peptide [31].

It was described earlier that proper anionic counterions can facilitate the direct penetration of octaarginine (Arg_8_) [32]. The best counter ion was the pyrenebutyric acid, which increased the direct delivery of GFP protein into HeLa cells. This compound was used as an additive, and it was not covalently attached to the CPP. Later, it was proposed that there is repulsion-driven ion-pairing interaction that accounts for the cellular action of the arginine-rich CPPs [33]. This process has been explained with the electron-rich pyrene surface, which helps to orientate the resulting CPP complex toward the interior of the membrane [34]. This molecule also has aromatic systems like the Dabcyl does.

In this work, we aimed to introduce a new 1-pyrene carboxylic acid (PCA) modification on the *N*-terminus and/or into a side chain. We determined the cellular uptake of different peptides and compared the influence of PCA on internalization with the effectiveness of the Dabcyl group. Furthermore, peptides containing tryptophan and PCA were investigated, too. We assume that this additional large hydrophobic group can help the peptides internalize better, and thus, this modification can lead us to new reliable transport molecules.

## 2. Results

### 2.1. Synthesis of Peptides

**Figure 1:**
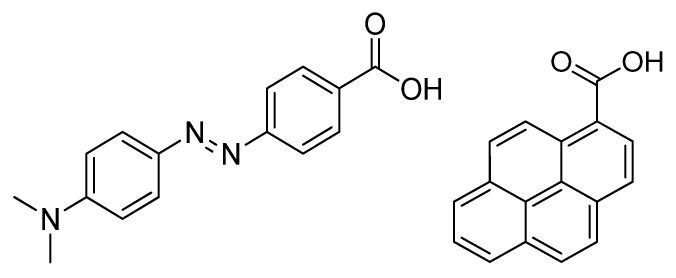
Structure of the Dabcyl group (**left**) and PCA group (**right**) used as novel modification.

In this work, oligoarginines were designed with a novel modification on the side chain or *N*-terminal amino group to study its effect on internalization. Peptides that contained Trp to increase hydrophobicity were also investigated. All peptide was synthesized manually by Fmoc/^t^Bu strategy using DIC and Oxima Pure as coupling reagents. The *N*-terminal or the side chain of peptides was modified by PCA (Figure 1) or the Dabcyl group. While the *N*-terminal modification was performed on the resin, the coupling to the side chain was carried out in solution using DIC, Oxima Pure, and DIEA. For the internalization studies, the peptides were fluorescent labeling by 5(6)-carboxyfluorescein (Cf) dye. The DauSuc as antitumor drug was attached to the peptide in solution.

The chemical characterization of the peptides and conjugates was performed using ESI-MS and analytical RP-HPLC (Table 1). Analytical RP-HPLC chromatograms and MS spectra are summarized in the Supplementary Materials, Figures S1–S30.

### 2.2. Cellular Uptake

**Figure 2:**
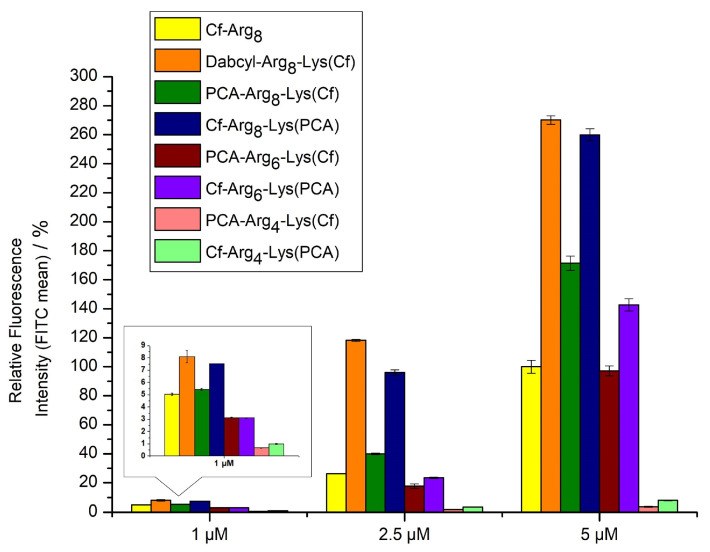
Cellular uptake of different oligoarginines on MDA-MB-231 cells. Peptides were measured at 1, 2.5 and 5 µM concentrations, where the cells were treated for 90 min. at 37 °C. The fluorescence intensities of cells were measured by flow cytometry. Fluorescence intensities are relative to Cf-Arg_8_ at 5 μM (100%). Data represents the mean ± standard deviation (SD).

The study of internalization was carried out on MDA-MB-231 cells by flow cytometry. The concentrations of labeled peptides were 1, 2.5, and 5 µM. The cells were treated for 90 min at 37 °C. Octaarginine was used as a positive control, and its cellular uptake was 100% at 5 µM concentration. The peptides were not toxic even in the highest concentration (Supplementary Materials, Figure S33). The peptides showed concentration-dependent uptake. The internalization of Dabcyl-Arg_8_-Lys(Cf) was the highest. Cf-Arg_8_-Lys(PCA) showed nearly similar internalization, almost 3-fold better than octaarginine. Surprisingly peptide PCA-Arg_8_-Lys(Cf) had weaker internalization but it still was 1.5 fold better than octaarginine (Figure 2). The hexaarginine derivatives had the same or better internalization than those of octaarginine, but in cases of four arginine residues, the PCA could not enhance the cellular uptake. However, the position of PCA has an influence on the uptake.

**Figure 3:**
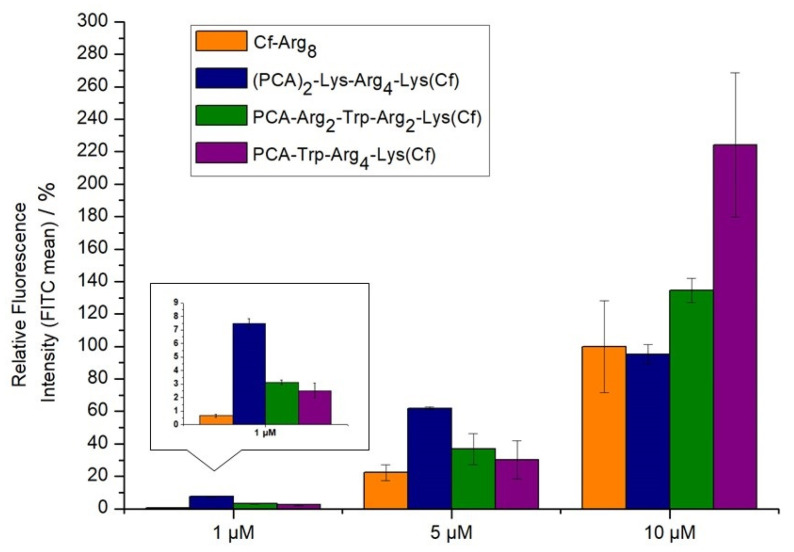
Cellular uptake of different oligoarginines on MDA-MB-231. Peptides were measured at 1, 5, and 10 µM concentrations where the cells were treated for 90 min. at 37 °C. The fluorescence intensities of cells were measured by flow cytometry. Fluorescence intensities are relative to Cf-Arg_8_ at 5 μM (100%). Data represents the mean ± standard deviation (SD).

The introduction of tryptophan into the sequence caused a dramatic effect on the cellular uptake of tetraarginine derivatives. PCA-Trp-Arg_4_-Lys(Cf) was two-fold better than octaarginine, while PCA-Arg2-Trp-Arg2-Lys(Cf) was only slightly better, indicating the importance of the Trp position as well (Figure 3). A peptide with two PCA-groups on the *N*-terminus was also examined. The (PCA)_2_-Lys-Arg_4_-Lys(Cf) derivate had the highest internalization at low concentrations, especially in 1 µM (Figure 3). Its activity is comparable with peptide Dabcyl-Arg_8_-Lys(Cf) and Cf-Arg_8_-Lys(PCA). Nevertheless, at higher concentrations, it loses the advantage of its efficacy. The peptides were not toxic even in the highest concentration (Supplementary Materials, Figure S34).

### 2.3. Investigation of Endocytic Pathways of Entry

**Figure 4:**
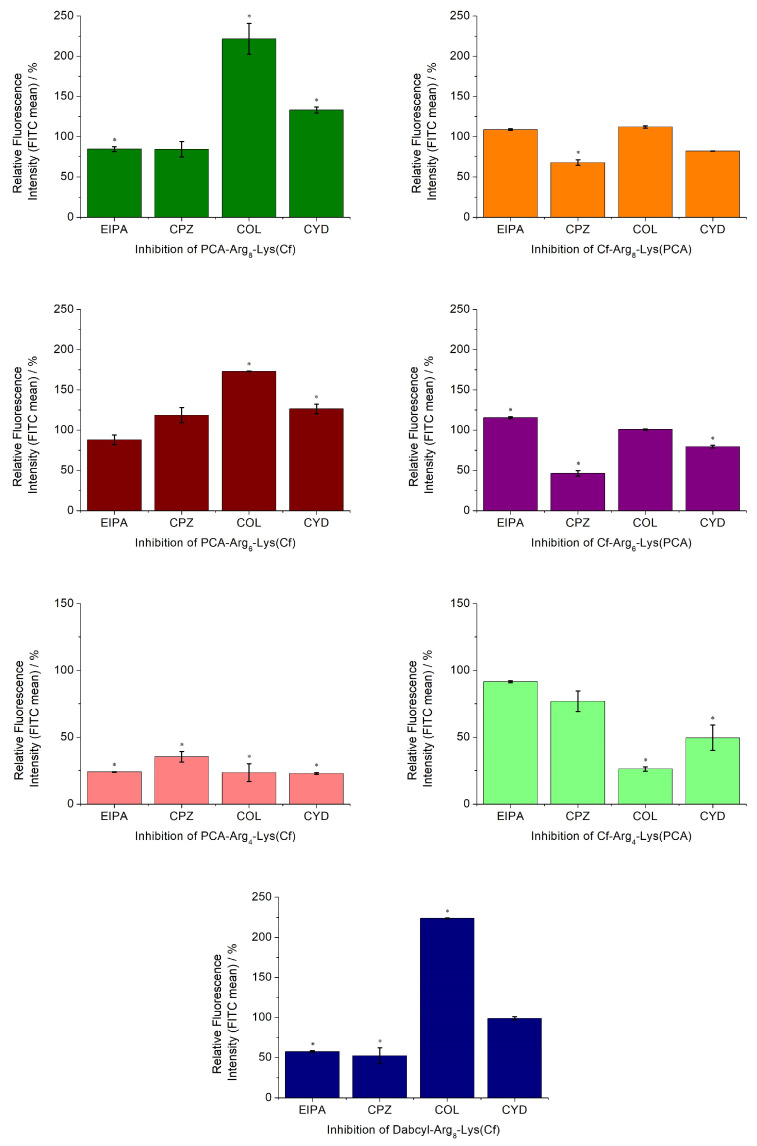
Effect of endocytosis inhibitors on the cellular uptake of peptides. The MDA-MB-231 cells were pretreated with the inhibitors EIPA (50 μM), CPZ (30 μM), CyD (5 mM), and COL (10 mM) for 30 min before the treatment with the peptide conjugates (5 μM) for 90 min. Student’s *t*-test (* *p* < 0.05) determined a significant difference from the control. Data represents the mean ± standard deviation (SD).

Peptides can use different entry pathways during their internalization. Different pathways were investigated using appropriate inhibitors (Figure 4). The caveolae/lipid-raft-mediated endocytosis was inhibited by methyl-β-cyclodextrin (CyD) [35], while macropinocytosis was inhibited by 5-(N-ethyl-N-isopropyl)amiloride (EIPA) [36]. The role of microtubules and clathrin-mediated endocytosis was studied using colchicine (Col) [37] and chlorpromazine (CPZ) [38], respectively.

Based on the effect of different inhibitors, the peptides can be grouped. The number of arginine residues and the position of PCA had the highest influence on the internalization pathways. The hexa- and octaarginine showed similar behavior, which depended on the position of PCA. In the case of *N*-terminal PCA modification, none of the inhibitors caused a reduction in the cellular uptake. Moreover, the inhibition of the microtubular system resulted in a significant increase in their internalization. The change in the position of PCA (put it onto the *C*-terminal Lys side chain) altered the picture. The uptake of these two derivatives was strongly dependent on the inhibition of clathrin-mediated endocytosis. The cellular uptake of these two peptides was also inhibited by CYD, the caveolae/lipid-raft-mediated endocytosis inhibitor, although the extent of this inhibition was lower. The behavior of tetraarginine derivatives was very different but also strongly dependent on the PCA position. All inhibitors diminished the internalization of peptides. The isomer derivative Cf-Arg_4_-Lys(PCA) showed clathrin-mediated and, to a lesser extent, caveolae/lipid-raft-mediated endocytosis-mediated internalization. The positive control peptide Dabcyl-Arg_8_-Lys(Cf) had similar behavior to those of PCA-Arg_6-8_-Lys(Cf), with only one difference. Its uptake was also mediated by macropinocytosis.

### 2.4. Intracellular Distribution of Peptides

**Figure 5:**
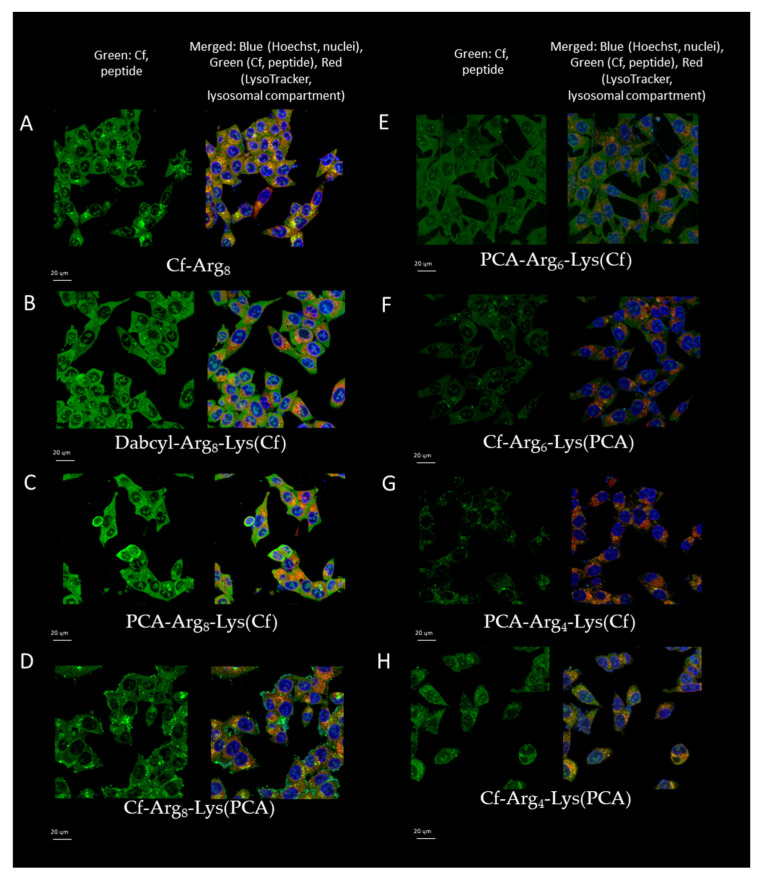
Intracellular localization of peptides measured by confocal microscopy. ((**A**): Cf-Arg_8_, (**B**): Dabcyl-Arg_8_-Lys(Cf), (**C**): PCA-Arg_8_-Lys(Cf), (**D**): Cf-Arg_8_-Lys(PCA), (**E**): PCA-Arg_6_-Lys(Cf), (**F**): Cf-Arg_6_-Lys(PCA), (**G**): PCA-Arg_4_-Lys(Cf), (**H**): Cf-Arg_4_-Lys(PCA)) MDA-MB-231 cells were treated with peptide conjugates (5 μM) for 90 min. The scale bar represents 20 µm.

Confocal laser scanning microscopy images were captured to assess intracellular distribution of the peptides (Dabcyl-Arg_8_-Lys(Cf), Cf-Arg_8,_ PCA-Arg_8_-Lys(Cf), Cf-Arg_8_-Lys(PCA), PCA-Arg_6_-Lys(Cf), Cf-Arg_6_-Lys(PCA), PCA-Arg_4_-Lys(Cf), Cf-Arg_4_-Lys(PCA)). After treatment, LysoTracker Deep Red was used for lysosome and Hoechst 33,342 for nuclear staining to distinguish subcellular localization of the peptides. The experiment was carried out after 90 min of incubation, and representative images are presented in (Figure 5). Based on the results of the flow cytometry measurements, all peptides were internalized efficiently. In all cases, fluorescent signals were observed mainly in lysosomal compartments (Figure 5). Peptides could be imaged in the cytosol with different levels of co-localization with lysosomal staining. Considering all these data, it was presumed that the peptides enter the cells concentration-dependent by direct penetration or endocytosis (Figure 5). The co-occurrence may subjectively identify the co-localization of Cf-peptide and LysoTracker. This simple spatial overlap is the combined contribution of both signals (green and red) when the images of each signal are superimposed (merged, see Supplementary Figures S31 and S32). Our main goal was to visualize the differences, and our experiments were mainly designed to compare qualitative data (Images with the labeling are in the Supplementary Materials, Figures S31–S32, Colocalization analysis Figures S35–S58, Tables S1–S24).

Cf-Arg_8_ mainly accumulated in lysosomal compartments (based on lysosomal staining). The octaarginine also had diffuse cytosolic distribution in a good correlation with earlier data at this concentration, and its cellular uptake is driven mainly by endocytosis via macropinocytosis (Figure 5). Strong co-localization was observed with lysosomes and just a minimal nucleus staining. In contrast, the *N*-terminal Dabcyl or PCA-modified octaarginine (PCA-Arg_8_-Lys(Cf) and Dabcyl-Arg_8_-Lys(Cf)) showed intensive diffuse distribution alongside minimal nuclear and lysosomal appearance. Peptide Cf-Arg_8_-Lys(PCA) had very different cellular distribution than those of peptide PCA-Arg_8_-Lys(Cf) and Dabcyl-Arg_8_-Lys(Cf). It had a lysosomal distribution, as in the case of CF-labeled octaarginine, although there was only a low diffuse fluorescence signal. In the case of shorter oligoarginines (hexa- and tetraarginine), the intracellular distribution was mainly vesicular punctuated with only one exception—PCA-Arg_6_-Lys(Cf), which showed diffuse distribution (Figure 5); this peptide was imaged in the cytosol with low level of co-localisation with lysosomal staining, which suggests that the peptide internalize and display a ubiquitous distribution in the cytosol. It is suggested that no vesicular transport is involved in the peptide uptake (no direct co-localization with lysosomes).

**Figure 6:**
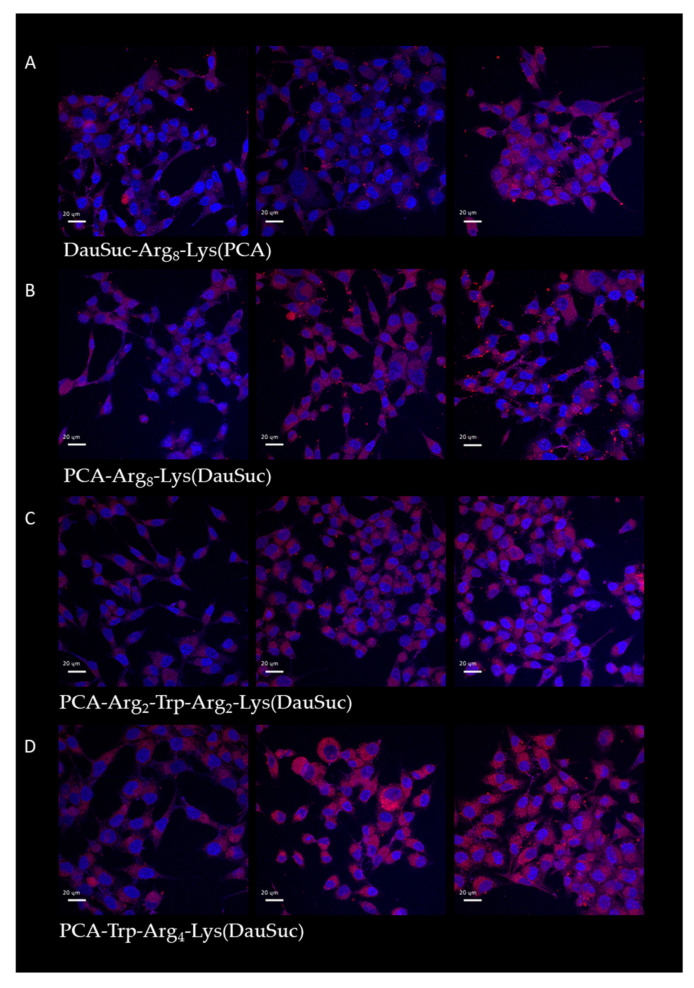
Intracellular localization of peptides with DauSuc measured by confocal microscopy. ((**A**): DauSuc-Arg_8_-Lys(PCA), (**B**): PCA-Arg_8_-Lys(DauSuc), (**C**): PCA-Arg_2_-Trp-Arg_2_-Lys(DauSuc), (**D**): PCA-Trp-Arg_4_-Lys(DauSuc)) MDA-MB-231 cells were treated with peptide conjugates (5 μM) for 15, 45, and 90 min. The scale bar represents 20 µm.

To employ the peptides for drug delivery, we used daunomycin as cargo. Its advantage is that it has autofluorescence property, which allows us to examine its cellular distribution without additional fluorescence labeling (Figure 6).

The cells’ fluorescence was monitored after 15, 45 and 90 min treatment. All the conjugates showed increased cellular uptake when the incubation time was extended to 45 min, but their intracellular amount did not change significantly after an additional 45 min. Peptides with PCA in their side chains had the highest internalization and conjugates with *N*-terminal PCA showed weaker fluorescence.

### 2.5. In Vitro Cytostatic Effect of Conjugates

Conjugates of the best peptides (PCA-Arg_8_-Lys, Arg_8_-Lys(PCA), PCA-Trp-Arg_4_-Lys, PCA-Arg_2_-Trp-Arg_2_-Lys) based on their cellular uptake were synthesized using a small drug molecule as a cargo. The peptides were conjugated with DauSuc, an antitumor drug in solution. In the conjugates, the DauSuc molecule was attached to the *N*-terminus or the ε-amino group of lysine at the *C*-terminus. The in vitro cytostatic activity was measured on MDA-MB-231 cells (Table 2).

All conjugates could inhibit the growth of tumor cells. Three of them have very close IC_50_ values (23.7–28.1 µM), and only one showed significantly weaker activity (84.0 µM).

## 3. Discussion

Oligoarginines, octa-, nona-, and decaarginine, are well known and commonly used cell-penetrating peptides used alone or in various drug-delivery constructs [39]. Unfortunately, shorter oligoarginines do not show effective internalization. Many trials have been conducted to modify longer oligoarginines to improve their internalization [17]. We hypothesize that short efficient oligoarginines may have many advantages over longer ones, such as simpler synthesis with lower costs and less possible interaction with the cargo molecules that are important in drug development for clinical use. Therefore, we are focusing on developing short oligoarginines with enhanced internalization properties by modifications with aromatic moieties. Our first finding was that the Dabcyl group may increase the internalization of hexa- and tetraarginine [30]. Although the latter had lower cellular uptake, it showed remarkable direct penetration, mainly at low concentrations. Thus, further modifications with Trp, amino benzoic acid, or aminomethyl benzoic acid were investigated [31,40]. In this work, the effect of PCA instead of Dabcyl group was examined on cellular uptake of shorter oligoarginines.

**Figure 7:**
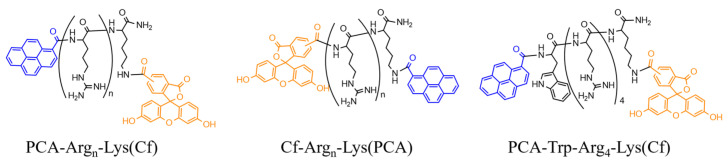
Structures of the different peptide derivatives.

In the first set of peptides, we examined the influence of PCA’s position on cellular uptake. The easiest way to modify CPP is on the *N*-terminal amino group. It can often be performed on the resin, although the modification may have a different effect if its position differs [41]. Thus, in some constructs, a C-terminal Lys residue was introduced into the sequence to carry the *C*-terminal PCA group (Cf-Arg_4_-Lys(PCA), Cf-Arg_6_-Lys(PCA), and Cf-Arg_8_-Lys(PCA)) (Figure 7). As Trp insertion increased dramatically, the cellular uptake of Dabcyl-modified tetraarginine PCA-modified derivatives with Trp residue were synthesized too (PCA-Trp-Arg_4_-Lys(Cf) and PCA-Arg_2_-Trp-Arg_2_-Lys(Cf)) (Figure 7).

The PCA-modified tetraarginines showed weak internalization compared to octaarginine (Figure 2). Their cellular uptake was low, regardless of the PCA group’s position. These results correlate with our earlier findings that this modification is not enough for efficient cell penetration in the case of Arg_4_ [30]. The effect of PCA group on the cellular uptake of hexaarginine (PCA-Arg_6_-Lys(Cf) and Cf-Arg_6_-Lys(PCA)) was similar to that of Dabcyl group. (Figure 2) [30]. These derivatives had identical or better cellular uptake than the CF-labeled octaarginine. Their internalization depends on the position of PCA, it had higher activity at the *C*-terminus.

When the effect of Trp on the internalization of tetraarginine was examined, similar results were noticed, like in our earlier study with combined Dabcyl and Trp modification [31]. One tryptophane was enough to dramatically increase the internalization of PCA-modified tetraarginine derivatives (Figure 3). In some concentrations, the position of Trp (in the middle or at the *N*-terminus of sequence) influenced the cellular uptake. Still, all derivatives had the same or better (2-fold) internalization than the CF-Arg_8_. The synergistic effect of PCA and tryptophane may result in an increase in aromaticity and hydrophobicity. It is supported by the other derivative with two PCA groups on the *N*-terminus ((PCA)_2_-Lys-Arg_4_-Lys(Cf)). This derivative had around the same internalization as CF-Arg_8_ but was much better at low concentration (1 µM) (Figure 3).

**Figure 8:**
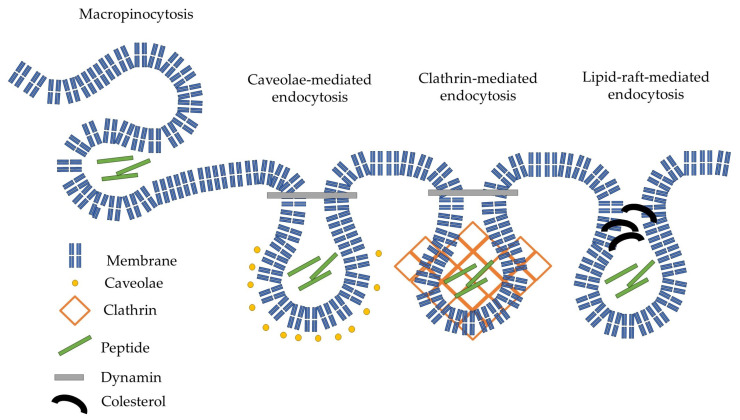
Schematic representation of the possible endocytic internalization of our peptides.

The effect of different inhibitors on the mechanism of internalization depended on the number of arginine residues and the position of PCA (Figure 4). The cellular uptake of longer arginine derivatives (hexa- and octaarginine) was or was not inhibited by any inhibitors in the same way. In the case of *N*-terminal modification with CPA, there was no inhibition, which means none of the examined pathways or intracellular components took significant parts in the internalization. The picture was the opposite if the PCA modification was carried out on the ε-amino group of *C*-terminal lysine. Some endocytic pathways became important for the internalization. One possible explanation of oligoarginine internalization is repulsion-driven ion-pairing interactions [33]. This interaction may improve using pyrene butyric acid as an activator [32]. The proposed basis of this activation ability is the ionpair-π interaction [33]. The long side chain of lysine may allow the PCA group to obtain a position in which it can interact with the guanidino group of the arginine side chain. This interaction somehow decreases the rate of direct internalization, and thus, the endocytic route becomes more pronounced. The effect of the PCA position on the cellular uptake of the tetra arginine derivative is more interesting (Figure 8).

In the case of the *N*-terminal position, all inhibitors blocked the internalization, revealing that the four guanidino groups could not induce direct penetration, and the internalization may happen via endocytosis. These and the value of the cellular uptake suggest that the main factor in the internalization is the interaction of arginine side chains with the membrane and PCA may enhance the internalization as hydrophobic/aromatic moiety (*N*-terminal modification) or via ionpair-π interaction. In the last case, it increases the degree of endocytosis. The importance of hydrophobic/aromatic properties of PCA in the case of tetraarginine is proven by the enhanced internalization of those derivatives with two PCA or one PCA and a Trp. The dependence of aromatic moieties on internalization efficiency is well correlated with earlier results about the possible balance between arginine residues and hydrophobicity [41]. Although octaarginine was reported to enter cells mainly by clathrin-mediated endocytosis [42,43], other sources suggested that macropinocytosis is the primary route [44,45]. None of our novel constructs showed similar inhibitor dependence in the internalization. This observation suggests that the PCA alters the cellular uptake route independent of its position in the constructs.

The role of endocytosis in the internalization described here is in a good relationship with the intracellular distribution of the peptides (Figure 5 and Figure 6). In the case of peptides showing endocytosis-mediated internalization, the distribution of the fluorescence signal was mainly diffuse, while the others had punctuated and diffused fluorescence signal. This kind of distribution was dependent on the position of PCA group. The same difference was observed between the two forms of peptides (*N*- or *C*-terminal position) in the distribution as in the effect of inhibitors. However, the main localization of peptides was in the cytosol, and they also showed signals in some regions of the nucleus, with one exception—PCA-Arg_4_-Lys(Cf) (Figure 5G).

The best constructs were conjugated with DauSuc (PCA-Trp-Arg_4_-Lys(DauSuc), PCA-Arg_2_-Trp-Arg_2_-Lys(DauSuc), PCA-Arg_8_-Lys(DauSuc), and DauSuc-Arg_8_-Lys(PCA)). All conjugates have moderate cytostatic effects (IC_50_ ~ 23–28 µM, Table 2). These results correlate well with earlier data where Dabcyl was used instead of PCA [31]. The only one exception was the PCA-Trp-Arg_4_-Lys(DauSuc) (IC_50_ ~ 84 µM). Although, this conjugate showed very efficient internalization. Based on its efficient cell permeability, our idea is that the possible interaction of the aromatic groups of the peptide and Dau may result in decreased activity.

## 4. Materials and Methods

### 4.1. Synthesis of Peptides and Their Conjugates

Peptides were synthesized manually using solid phase peptide synthesis with the Fmoc/^t^Bu strategy on Rink amide MBHA resin. The side chain functional group of amino acid residues was protected as follows: lysine by tert-butyloxycarbonyl (Boc) group and arginine by the 2,2,4,6,7-pentamethyldihydrobenzofuran-5-sulfonyl (Pbf) group. The Fmoc group, which protects the α-amino group of amino acids, was removed by the solution of 2% 1,8-diazabicycloundec-7-ene (DBU) and 2% piperidine in dimethyl formamide (DMF). This deprotection step took 2 + 2 + 5 + 10 min. The coupling of the amino acid residues were carried out in DMF with 3 eq of the amino acid derivative N,N′-diisopropylcarbodiimide (DIC) and ethyl cyano(hydroxyimino)acetate (Oxima Pure) for 1 h at RT. Then, the resin was washed with DMF (3 × 1 min) and with DCM (3 × 1 min). The coupling of the amino acids was monitored by the Kaiser Test. In case of a positive result, the amino acid was recoupled (once or twice) with the same parameters. When the last amino acid was coupled with the *N*-terminal modification, Dabcyl, Cf (5(6)-carboxyfluorescein), or PCA was attached similarly to the amino acids. In the case of the peptide DauSuc-Arg_8_-Lys(PCA), the lysine residue was protected with the 1-(4,4-dimethyl-2,6-dioxocyclohex-1-ylidene)ethyl (Dde) group on its side chain for selective removal. In this case, after the removal of the last Fmoc group, the Boc protecting group was coupled to the *N*-terminus using (Boc)_2_O reagent, and then the Dde group was removed using a solution of 2% hydrazine in DMF for 5 + 5 + 5 + 5 + 5 + 5 min. After this, PCA was coupled to the lysine side chain. For the synthesis of (PCA)_2_-Lys-Arg_4_-Lys(Cf), di-Fmoc-protected lysine (Fmoc-Lys(Fmoc)-OH) was used. When it was deprotected, two PCA groups were coupled. After the finished sequence, peptides were cleaved from the resin by a solution of 5 mL TFA containing 0.365 g phenol, 0.25 mL distilled water, 0.25 mL thioanisole, and 0.125 mL TIS as scavengers for 3 h at RT. After the cleavage, the peptides were precipitated with diethyl–ether. The crude product was dissolved in 10% acetic acid and was lyophilized. Semi-preparative RP-HPLC was applied to purify the peptides, and they were characterized using analytical RP-HPLC and ESI-MS. The purity of the compounds was higher than 90%. In some cases, the isomers of Cf could be separated by the analytical HPLC providing a peak not related to impurities.

The purified peptides were modified in solution on their Lys side chain by CF, PCA, or succinylated daunomycin (DauSuc). There was one exception, DauSuc-Arg_8_-Lys(PCA), in which the *N*-terminus was reacted with DauSuc. Peptides were reacted with 1 eq. of dye, PCA or drug, 1 eq. of DIC and Oxima Pure and 3 eq. of N,N′-diisopropylethylamine (DIEA) for one day at RT. Then, the reaction mixtures were purified by RP-HPLC and characterized by analytical RP-HPLC and ESI-MS.

### 4.2. Flow Cytometry

#### MDA-MB-231

The cellular uptake of fluorescently labeled peptides was measured using MDA-MB-231 (ATCC: HTB-26) human triple-negative breast adenocarcinoma cells. Before the treatment the cells were grown on 24-well with 10^5^ cells per well. Cells treated by serum free medium were the negative controls. Different peptide solutions were used in the experiments, and the treatment was conducted for 90 min at 37 °C. The activity of peptides was measured in different concentrations 1, 2.5, 5 μM. After the incubation, 100 μL trypsin (0.25%) was applied to detach the cells and to cleave excess and non-internalized peptides for 5–10 min. After the trypsinization, 800 µL of HPMI buffer (glucose, NaHCO_3_, NaCl, HEPES, KCl, MgCl_2_, CaCl_2_, Na_2_HPO_4_ × 2 H_2_O) containing 10% fetal bovine serum (FBS) was added to halt the activity of trypsin. The cells were put into falcon tubes and then were centrifuged at 216× *g* at 4 °C. The supernatant was removed, and the cells were suspended in 250 μL HPMI. The fluorescence intensity of cells was measured by flow cytometer (BDLSR II, BD Bioscience, San Jose, CA, USA). Data were analyzed with FACSDiVa 5.0 software (BD Bioscience, San Jose, CA, USA).

Inhibitors were administered to investigate the internalization mechanism of the peptides. To inhibit caveolae/lipid-raft-mediated endocytosis methyl-β-cyclodextrin (CyD) [35], to investigate the role of microtubules colchicine (Col) [37], and to inhibit micropinocytosis, 5-(N-ethyl-N-isopropyl)amiloride (EIPA) was applied [36], and clathrin-mediated endocytosis was inhibited with chlorpromazine (CPZ) [38]. First, the cells were treated only with the inhibitors for 30 min, and then the peptide conjugates were added at 5 µM. After this, the cells were incubated at 37 °C for 90 min.

### 4.3. Analysis of In Vitro Cytostatic Activity of Conjugates

The in vitro measurements were carried out on 96-well plates containing 5 × 10^3^ MDA-MB-231 cells per well. The cells were incubated for 24 h at 37 °C. The measurement started with treating these cells with the solutions of compounds for 3 h, where the final volume was 200 μL. The cytostatic activity was measured on a broad spectrum of concentrations between 0.000128 and 100 μM. Serum-free medium was used on cells for 3 h at 37 °C as control. After treatment, the compounds were washed out using a serum-free medium twice. The cells were then cultured for 72 h in a medium with serum. Alamar-blue (Resazurim) assay was used to determine the ratio of live cells and thus the IC_50_. Briefly, Alamar-blue solution (22.5 μL, 0.15 mg/mL in PBS (pH = 7)) was added to the cells. Synergy H4 multimode microplate reader (BioTek, Winooski, VT, USA) was used to determine the fluorescence (at λ = 530/30 nm and 610/10 nm) after 4 h incubation. Cytostasis was calculated as Cytostatic effect (%) = [1 − (ODtreated/ODcontrol)] × 100; where ODtreated and ODcontrol correspond to the optical densities of the treated and untreated cells, respectively. Four parallel measurements were conducted in two independent experiments in each. Dose–response curves were used to determine the 50% inhibitory concentration (IC50). The curves were defined using MicrocalTM Origin (version 8.0, OriginLab, Northampton, MA, USA) software: cytostasis was plotted as a function of concentration, fitted to a sigmoidal curve, and the IC_50_ value was determined. IC_50_ represents the concentration of a compound required for 50% inhibition of growth in vitro and is expressed as micromolar units.

### 4.4. In Vitro Intracellular Localization Using Confocal Laser Scanning Microscopy (CLSM)

MDA-MB-231 cells were seeded in a complete medium in 24-well cell culture plates (Greiner Bio-One) with cover glasses (Assistant) for 24 h before the experiment (10^5^ cells/1 mL/well). Cells were treated with the Cf-PCA peptides (concentration: 5 µM) for 45 min in ICM. In the case of Dau-conjugates, cells were treated for 15, 45, and 90 min.

According to the manufacturer’s suggestions, the lysosomes were labeled by LysoTracker Deep Red; Hoechst 33,342 was used to stain the nuclei. Between the staining steps, cells were washed by ICM three times and by PBS two times before fixation. Cells were fixed with 4% PFA for 20 min at 37 °C and washed with PBS three times and distilled water twice. Cover glasses were mounted to microscopy slides (VWR) by Mowiol 4–88 mounting medium.

Confocal microscopy was performed on a Leica TCS SP8 Lightning Confocal Laser Scanning microscope (Leica, Wetzlar, Germany) with a 63× oil objective (parameters: Cf-PCA peptides λ_ex_ = 488 nm, λ_em_ = 541 nm, nuclei λ_ex_ = 405 nm, λ_em_ = 467 nm (Hoechst 33342), lysosomes λ_ex_ = 633 nm, λ_em_ = 720 nm (LysoTracker Deep Red)). Image J software (https://imagej.net/ij/download.html, 12 December 2024) was used for image processing.

### 4.5. Statistical Analysis

The value of cellular uptake was characterized by the mean value and standard deviation. The significancy was analyzed by Student’s *t*-test. Results were considered statistically significant if their *p*-values were less than 0.05.

## 5. Conclusions

In this work, different oligoarginines were synthesized and modified with the novel internalization enhancer PCA group, which was used in various positions (*N*- or *C*-terminal). The PCA group is a polycyclic aromatic group that can help the interaction of the peptides with the cell surface by increasing hydrophobicity. The effect of PCA was improved with the incorporation of tryptophane as an aromatic amino acid. These peptides exceeded the internalization of octaarginine, a well known CPP. The PCA position has a significant impact on internalization. The construct showed higher internalization when the PCA was attached to the side chain amino group of *C*-terminal Lys. The best conjugate, Cf-Arg_8_-Lys(PCA), internalized almost 3-fold better than the well-known octaarginine CPP. Although PCA increased the internalization of tetraarginine, its effectiveness is very far from that of the best conjugates. However, incorporating Trp residue into its sequence next to the PCA modification improved the cellular uptake, and very effective tetra arginine conjugates were synthesized. The position of PCA also changed the internalization pathway. Peptides with altered PCA positions showed different dependence on the effect of inhibitors of different endocytosis. In this work, we proved that the covalently attached pyrene carboxylic acid may enhance the cellular uptake of oligoarginines, and its effect can be improved using aromatic amino acid tryptophan.

PCA modification results in efficient cellular uptake via a different internalization pathway than Dabcyl derivatives. This chemical modification provides a new tool to develop effective drug delivery systems.

## Figures and Tables

**Table 1:** Chemical characterization of peptide conjugates.

Sequence	R_t_	M_calc_	M_meas_
Dabcyl-Arg_8_-Lys(Cf)	13.3	2003.08	2003.79
PCA-Arg_4_-Lys(Cf) ^a^	14.3	1355.63	1355.57
PCA-Arg_6_-Lys(Cf)	13.7	1667.83	1667.78
PCA-Arg_8_-Lys(Cf)	13.5	1980.04	1980.08
Cf-Arg_4_-Lys(PCA)	14.6	1355.63	1355.51
Cf-Arg_6_-Lys(PCA)	14.1	1667.83	1667.76
Cf-Arg_8_-Lys(PCA)	14.6	1980.04	1980.12
(PCA)_2_-Lys-Arg_4_-Lys(Cf)	16.2	1712.45	1712.77
PCA-Trp-Arg_4_-Lys(Cf)	15.0	1541.71	1541.43
PCA-Arg_2_-Trp-Arg_2_-Lys(Cf)	14.2	1541.71	1541.32
PCA-Trp-Arg_4_-Lys(DauSuc)	18.0	1792.65	1792.88
PCA-Arg_2_-Trp-Arg_2_-Lys(DauSuc)	16.4	1792.65	1792.91
PCA-Arg_8_-Lys(DauSuc)	15.3	2230.98	2231.44
DauSuc-Arg_8_-Lys(PCA) ^a^	15.5	2230.98	2231.36
Cf-Arg_8_	11.6	1624.14	1623.86

The purity analysis was performed by recording analytical chromatograms using Hypersil Hypurity C18 column (4.6 mm × 150 mm, 5 μm, 190 Å) with linear gradient elution (0 min 0% B, 2 min 0% B, 22 min 90% B) at 1 mL/min flow rate. The absorbance was measured at λ = 220 nm. Bruker Amazon SL (Bremen, Germany) mass spectrometer was used for the mass spectrometric analysis. The samples were dissolved in acetonitrile–water (50:50, *v*/*v*), containing 0.1% formic acid. ^a^ The purity of these peptides less than 90%.

**Table 2:** The cytostatic activity of peptide-drug conjugates on MDA-MB-231.

Conjugate	IC_50_ ± SD (µM) *
	MDA-MB-231
PCA-Arg_8_-Lys(DauSuc)	28.1 ± 7.5
DauSuc-Arg_8_-Lys(PCA)	23.7 ± 4.3
PCA-Trp-Arg_4_-Lys(DauSuc)	84.0 ± 7.3
PCA-Arg_2_-Trp-Arg_2_-Lys(DauSuc)	27.1 ± 6.0
DauSuc	>100 [31]

* The cells were incubated with the compounds for 3 h and cultured in a serum-containing medium for 3 days. The IC_50_ values were determined by Alamar-blue assay as described in the text. Standard deviation values (SD) are also presented.

## Data Availability

The original contributions presented in this study are included in the article/Supplementary Materials. Further inquiries can be directed to the corresponding author(s).

## Supplementary Materials

The following supporting information can be downloaded at: https://www.mdpi.com/article/10.3390/ijms26052202/s1. References [46,47,48,49,50,51,52,53,54,55] are cited in the Supplementary Materials.
