# Supplementary material for: 1-Pyrene Carboxylic Acid: An Internalization Enhancer for Short Oligoarginines
Source: Int J Mol Sci. 2025 Feb 28;26(5):2202. doi: 10.3390/ijms26052202 (PMC11900394; doi:10.3390/ijms26052202)
Supplement: Supplementary file 1 [file ijms-26-02202-s001.zip › ijms-3444848-supplementary.pdf]

International Journal of
Molecular Sciences

MDPI

Supplementary Materials: 1-Pyrene Carboxylic acid: An Internalization Enhancer for Short Oligoarginines

1. Materials

All amino acid derivatives, Fmoc-protected amino acids, N,N'-diisopropylcarbodiimide (DIC), and Rink-amide MBHA resin were purchased from IRIS BiotechGmbH (Marktredwitz, Germany). N,N-diisopropylethylamine (DIEA), 1.8-diazabicyclo[5.4.0]undec-7-ene (DBU), thioanisole were FLUKA (Buchs, Switzerland). Solvents for synthesis and purification were obtained from Molar Chemicals Ltd (Budapest, Hungary). 1-Pyrenecarboxylic acid (PCA), Ethyl cyano(hydroxyimino)acetate (Oxima Pure), phenol, 5(6)-carboxyfluorescein (Cf), trifluoroacetic acid (TFA), colchicine (COL), Resazurim (Alamar-blue) and all other chemicals used in biological experiments were purchased from Sigma Aldrich (Hungary). While tris(hydroxymethyl)aminomethane (TRIS) was purchased from VWR (Hungary). Dabcyl was purchased from AAT Bioquest. Chlorpromazine (CPZ), methyl-beta-cyclodextrin (CyD) were purchased from TCI chemicals.

For the *in vitro* assays DMEM medium, phosphate buffered saline (PBS) trypan blue, and L-glutamine were from Lonza (Basel, Switzerland). Pyruvate, trypsin, paraformaldehyde (PFA), Mowiol 4–88 were obtained from Sigma-Aldrich (Budapest, Hungary). Non-essential amino acids, fetal bovine serum (FBS) and Penicillin/Streptomycin (10,000 units penicillin and 10 mg streptomycin/mL) were from Gibco (Thermo Fisher Scientific, Waltham, MA, USA). HPMI buffer was prepared in our laboratory using components (glucose, $NaHCO_3$, NaCl, *N*-(2-hydroxyethyl)piperazine-*N′*-(2-ethanesulfonic acid - HEPES, KCl, $MgCl_2$, $CaCl_2$, $Na_2HPO_4 \times 2\ H_2O$) obtained from Sigma-Aldrich. Hoechst 33342 (62249), LysoTracker Deep Red (L12492, for fixed cells) were from Invitrogen Biotechnology (Thermo Fisher Scientific, Waltham, MA, USA).

1.1. Synthesis of succinylated daunomycin (DauSuc)

Inclusion of the succinyl linker into daunomycin was performed in a method reported earlier. [46] 100 mg daunomycin×HCl was reacted with 2 eq succinic anhydride in the presence of 3 eq DIEA, the reactants were allowed to mix overnight in 5 ml DMF. The solvent was subsequently evaporated, and the remaining crude product was dissolved in mobile phase B (0.1%TFA in 80% ACN:20% H2O) and further purified with RP-HPLC on a C18 Column.

1.2. RP-HPLC

Analytical RP-HPLC was performed on Exformma (Exformma Technology (ASIA) Co., Ltd, Hong Kong, China) HPLC system. The used column was Hypersil Hypurity C18 column (4.6 mm × 150 mm, 5 µm, 190 Å). Linear gradient elution (0 min 0% B; 2 min 0% B; 22 min 90% B) was used with eluent A (0.1% TFA in water) and eluent B (0.1% TFA in acetonitrile-water (80:20, v/v)) at 1mL/min flowrate, the peaks were detected at λ= 220 nm for both analytical and preparative RP-HPLC. The samples were dissolved in a minimum amount of eluent B and injected into the analytical RP-HPLC. The crude products were purified on a semi-preparative Phenomenex Jupiter C18 column (250 × 10 mm I.D.)

with 10 mm silica (300 Å pore size) (Torrance, CA, USA). Flow rate was 4 mL/min and linear gradient elution was applied. The samples were dissolved in eluent A containing small percentage of eluent B (10–25% depending on sequence).

1.3. Mass spectrometry

The molecular weight of peptides and conjugates was determined with ESI-MS using Bruker Amazon SL (Germany). The samples were dissolved in a water-acetonitrile solution (50:50) with 0.1% formic acid and directly injected with a syringe pump. Parameters: capillary voltage: 4 kV, nebulizer gas: 10 psi, dry gas: 4 L/min, heated capillary temperature: 250 °C.

1.4. Cell Culture

MDA-MB-231 [47] culture was a generous gift from Dr. József Tóvári (Department of Experimental Pharmacology and the National Tumor Biology Laboratory, National Institute of Oncology, Budapest, Hungary). The cells were cultured in DMEM supplemented with 10% FBS, 2 mM L-glutamine, 100 µg/mL Penicillin/Streptomycin, 1 mM Pyruvate and 1% non-essential amino acids (CM DMEM) using sterile T25 and T75 flasks with ventilation cap (Sarstedt, Nümbrecht, Germany) at 37 °C in a humidified atmosphere with 5% CO2 in ESCO Cell Culture Incubator (ESCO, Friedberg, Germany). Manipulations with the cells were performed in the laminar biosafety cabinet ESCO Sentinel Gold class II model AC2-4E8 (ESCO). No mycoplasma contamination was detected in the cell cultures.

1.5. Determination of in vitro cellular internalization

HPMI buffer (9 mM glucose, 10 mM NaHCO3, 119 mM NaCl, 9 mM HEPES, 5 mM KCl, 0.85 mM MgCl2, 0.053 mM CaCl2, 5 mM Na2HPO4 × 2 H2O, pH 7.4). [48]
The intracellular fluorescence intensity of cells was measured at λex = 488 nm (Coherent Sapphire laser excitation, emission channel—LP 510, BP 530/30). The results were analyzed with FACSDiva software.
In the case of Dau-conjugates, the intracellular fluorescence intensity of the cells was measured on channel PE LP550 (emission at λ = 550 nm), and data were analyzed with FACSDiva 5.0 software. All measurements were performed in triplicates.

2. Chemical Characterization of Peptide Conjugates

2.1. RP-HPLC

Analytical RP-HPLC was performed on Exformma (Exformma Technology (ASIA) Co., Ltd, Hong Kong, China) HPLC system. The used column was Hypersil Hypurity C18 column (4.6 mm × 150 mm, 5 µm, 190 Å). Linear gradient elution (0 min 0% B; 2 min 0% B; 22 min 90% B) was used with eluent A (0.1% TFA in water) and eluent B (0.1% TFA in acetonitrile-water (80:20, v/v)) at 1 mL/min flowrate, the peaks were detected at λ= 220 nm for both analytical and preparative RP-HPLC. The samples were dissolved in a minimum amount of eluent B and injected into the analytical RP-HPLC. The crude products were purified on a semi-preparative Phenomenex Jupiter C18 column (250 × 10 mm I.D.) with 10 mm silica (300 Å pore size) (Torrance, CA, USA). Flow rate was 4 mL/min and linear gradient elution was applied. The samples were dissolved in eluent A containing small percentage of eluent B (10–25% depending on sequence).

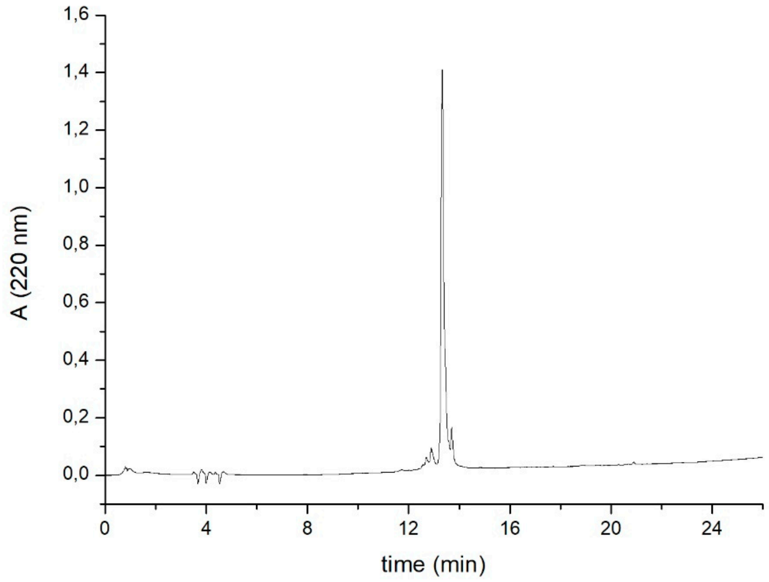

Figure S1. HPLC chromatogram of Dabcyl-Arg8-Lys(Cf). Retention time was obtained on Hypersil Hypurity C18 column (4.6 mm x 150 mm, 5 µm, 190 Å). The applied linear gradient elution was 0 min 0% B, 2 min 0% B, 22 min 90% B at 1 mL/min flow rate. The detection was carried on at $\lambda = 220$ nm.

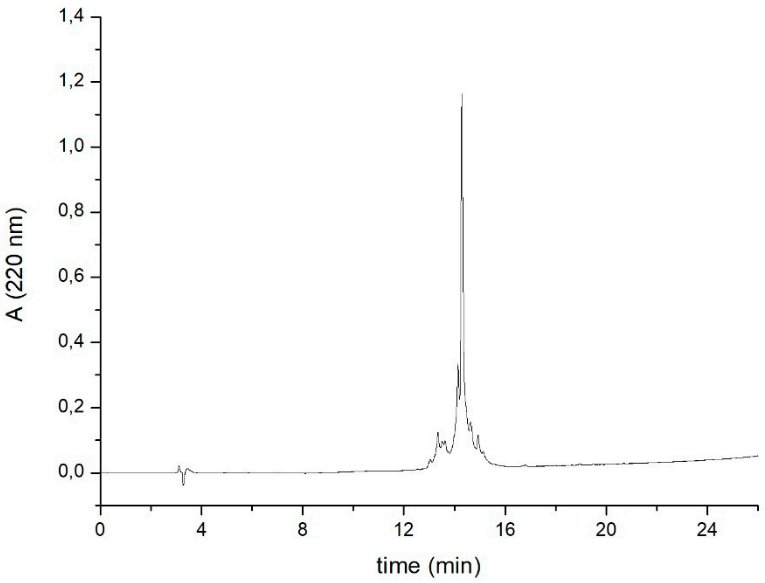

Figure S2. HPLC chromatogram of PCA-Arg4-Lys(Cf). Retention time was obtained on Hypersil Hypurity C18 column (4.6 mm x 150 mm, 5 µm, 190 Å). The applied linear gradient elution was 0 min 0% B, 2 min 0% B, 22 min 90% B at 1 mL/min flow rate. The detection was carried on at $\lambda = 220$ nm.

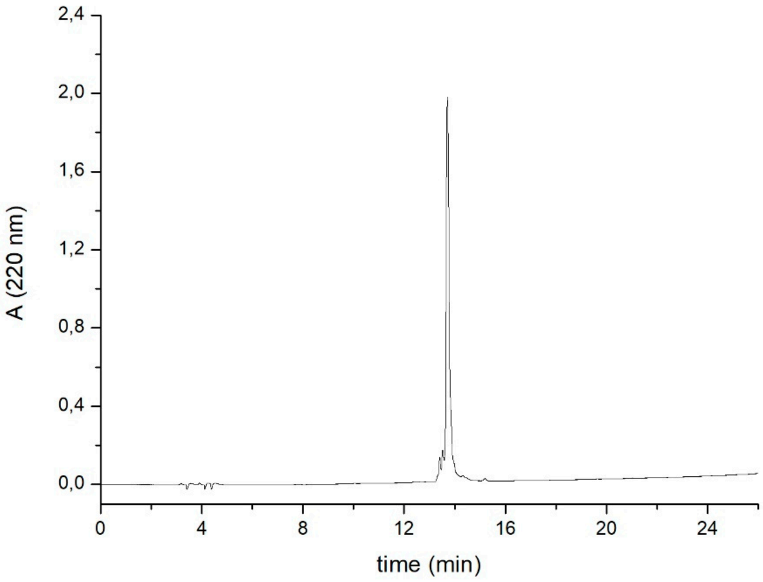

Figure S3. HPLC chromatogram of PCA-Arg$_6$-Lys(Cf). Retention time was obtained on Hypersil Hypurity C18 column (4.6 mm x 150 mm, 5 μm, 190 Å). The applied linear gradient elution was 0 min 0% B, 2 min 0% B, 22 min 90% B at 1 mL/min flow rate. The detection was carried on at λ = 220 nm.

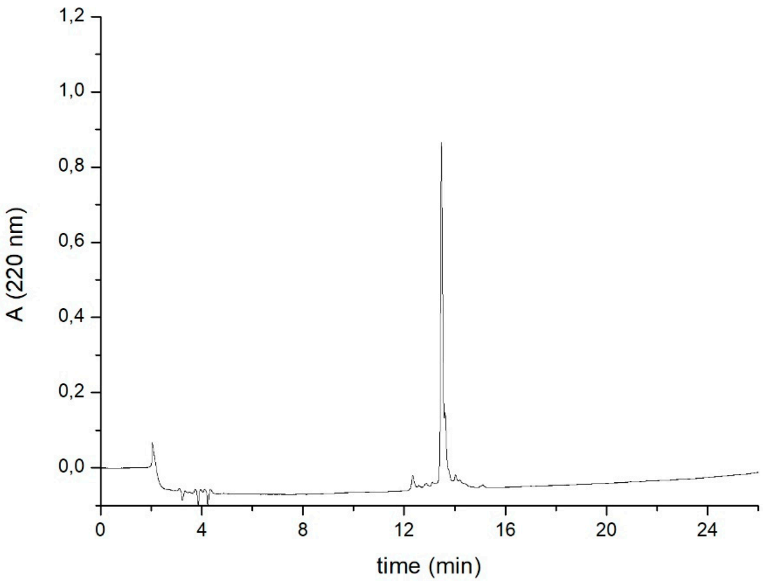

Figure S4. HPLC chromatogram of PCA-Arg$_8$-Lys(Cf). Retention time was obtained on Hypersil Hypurity C18 column (4.6 mm x 150 mm, 5 μm, 190 Å). The applied linear gradient elution was 0 min 0% B, 2 min 0% B, 22 min 90% B at 1 mL/min flow rate. The detection was carried on at λ = 220 nm.

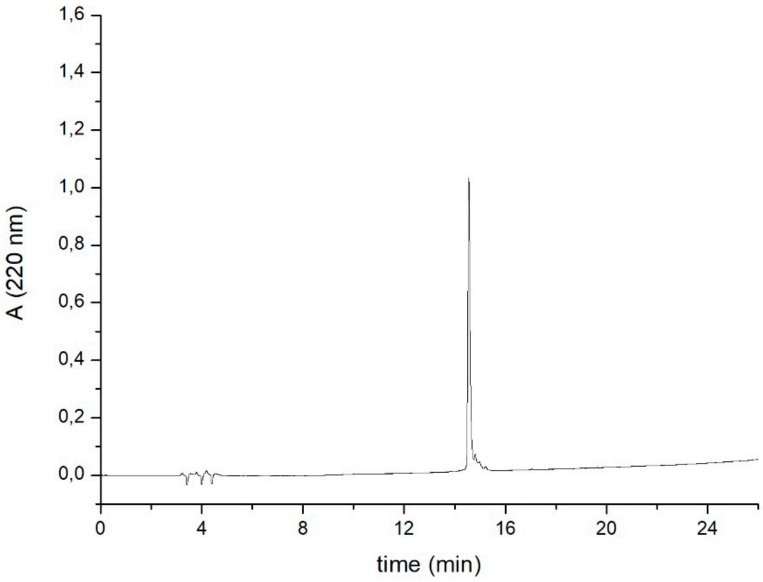

Figure S5. HPLC chromatogram of Cf-Arg$_4$-Lys(PCA). Retention time was obtained on Hypersil Hypurity C18 column (4.6 mm x 150 mm, 5 µm, 190 Å). The applied linear gradient elution was 0 min 0% B, 2 min 0% B, 22 min 90% B at 1 mL/min flow rate. The detection was carried on at λ = 220 nm.

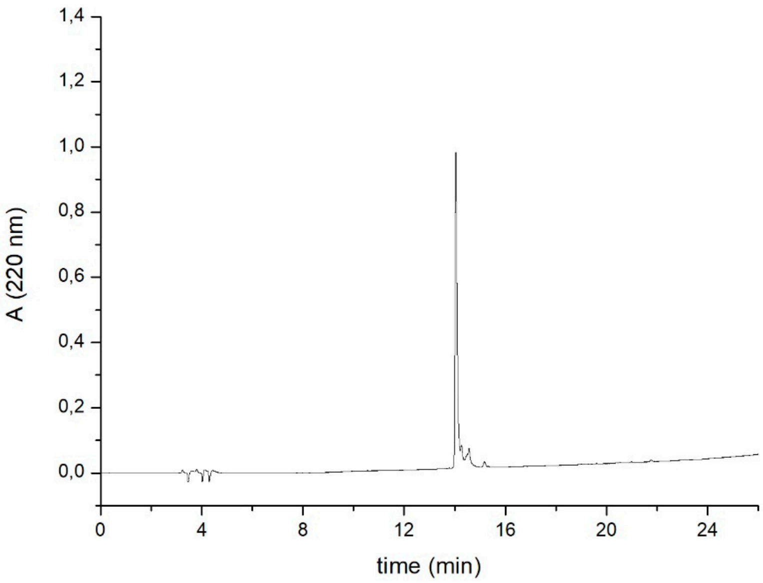

Figure S6. HPLC chromatogram of Cf-Arg$_6$-Lys(PCA). Retention time was obtained on Hypersil Hypurity C18 column (4.6 mm x 150 mm, 5 µm, 190 Å). The applied linear gradient elution was 0 min 0% B, 2 min 0% B, 22 min 90% B at 1 mL/min flow rate. The detection was carried on at λ = 220 nm.

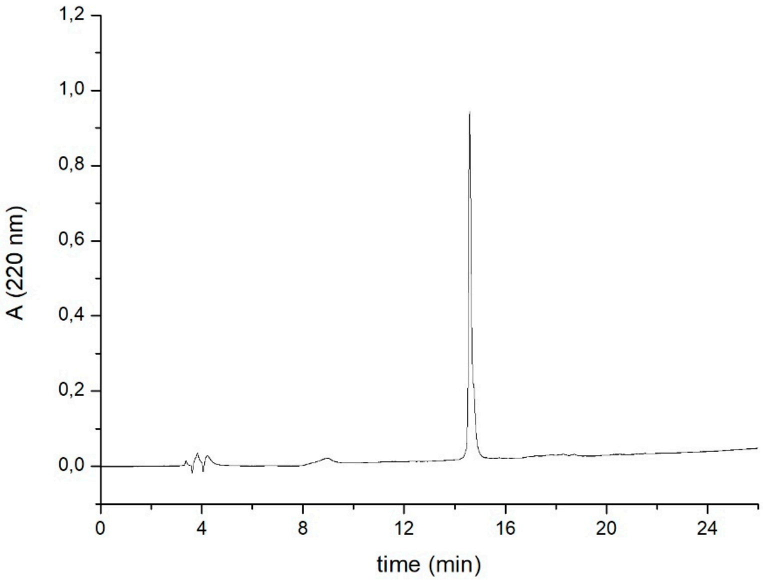

Figure S7. HPLC chromatogram of Cf-Arg_8-Lys(PCA). Retention time was obtained on Hypersil Hypurity C18 column (4.6 mm x 150 mm, 5 µm, 190 Å). The applied linear gradient elution was 0 min 0% B, 2 min 0% B, 22 min 90% B at 1 mL/min flow rate. The detection was carried on at λ = 220 nm.

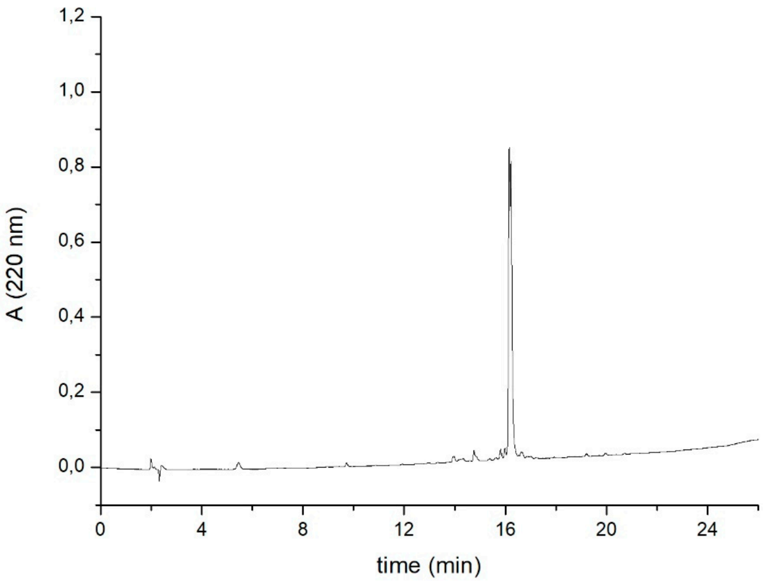

Figure S8. HPLC chromatogram of $(PCA)_2$-Lys-Arg_4-Lys(Cf). Retention time was obtained on Hypersil Hypurity C18 column (4.6 mm x 150 mm, 5 µm, 190 Å). The applied linear gradient elution was 0 min 0% B, 2 min 0% B, 22 min 90% B at 1 mL/min flow rate. The detection was carried on at λ = 220 nm.

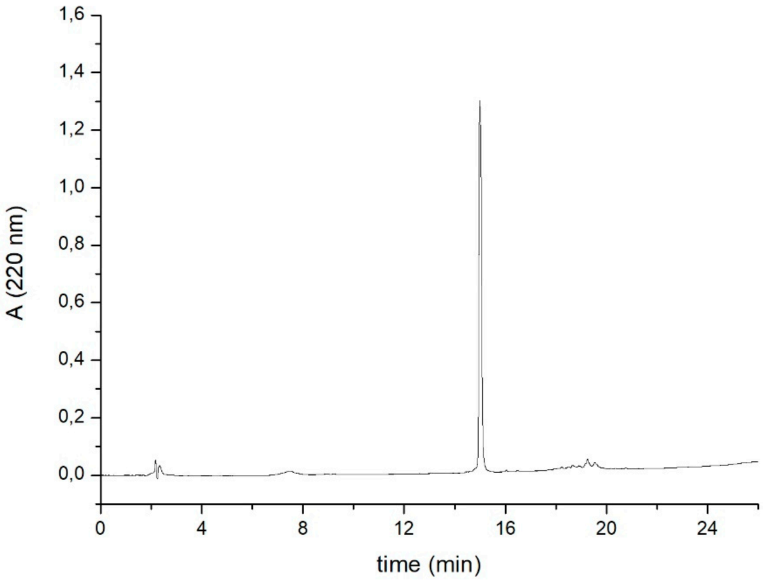

Figure S9. HPLC chromatogram of PCA-Trp-Arg_4-Lys(Cf). Retention time was obtained on Hypersil Hypurity C18 column (4.6 mm x 150 mm, 5 μm, 190 Å). The applied linear gradient elution was 0 min 0% B, 2 min 0% B, 22 min 90% B at 1 mL/min flow rate. The detection was carried on at λ = 220 nm.

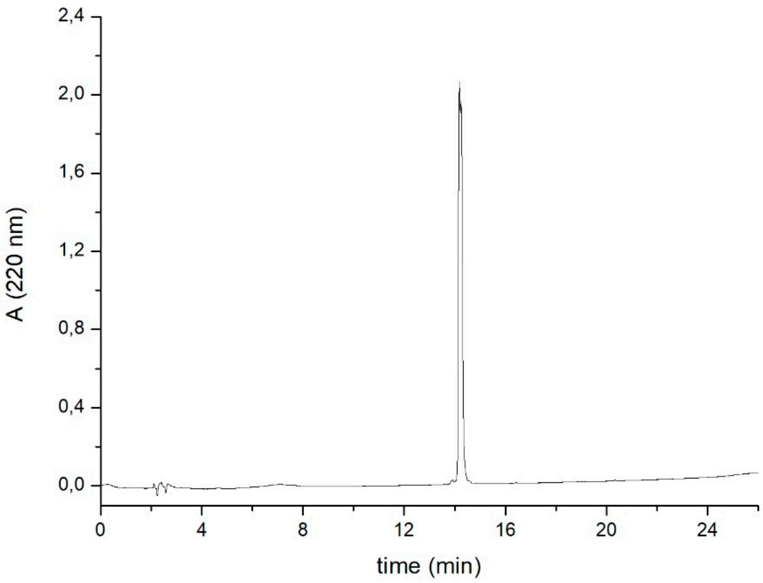

Figure S10. HPLC chromatogram of PCA-Arg_2-Trp-Arg_2-Lys(Cf). Retention time was obtained on Hypersil Hypurity C18 column (4.6 mm x 150 mm, 5 μm, 190 Å). The applied linear gradient elution was 0 min 0% B, 2 min 0% B, 22 min 90% B at 1 mL/min flow rate. The detection was carried on at λ = 220 nm.

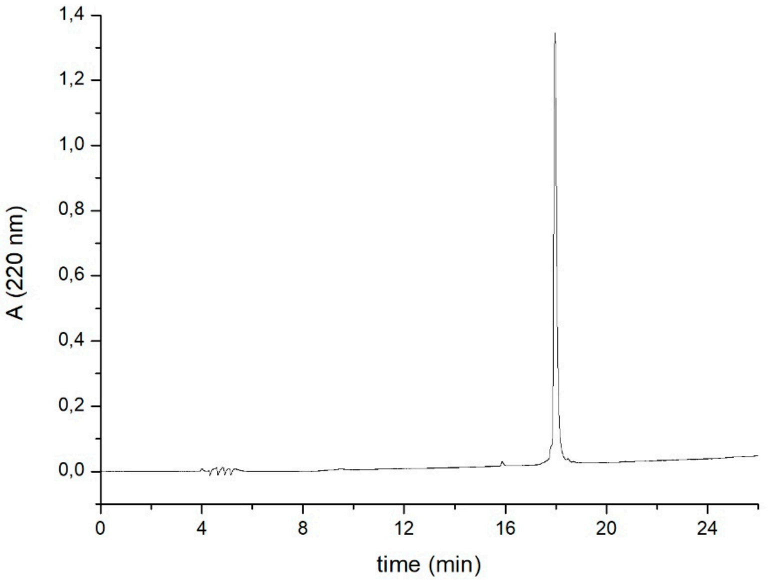

Figure S11. HPLC chromatogram of PCA-Trp-Arg4-Lys(DauSuc). Retention time was obtained on Hypersil Hypurity C18 column (4.6 mm x 150 mm, 5 μm, 190 Å). The applied linear gradient elution was 0 min 0% B, 2 min 0% B, 22 min 90% B at 1 mL/min flow rate. The detection was carried on at λ = 220 nm.

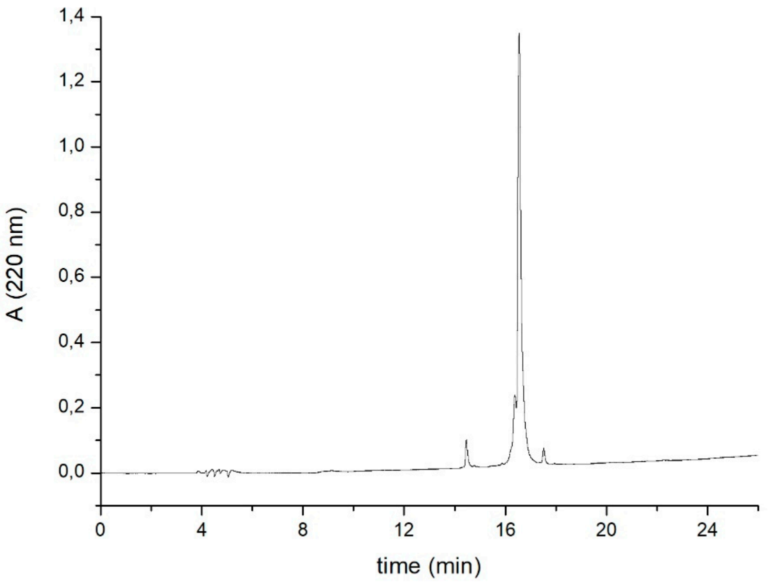

Figure S12. HPLC chromatogram of PCA-Arg2-Trp-Arg2-Lys(DauSuc). Retention time was obtained on Hypersil Hypurity C18 column (4.6 mm x 150 mm, 5 μm, 190 Å). The applied linear gradient elution was 0 min 0% B, 2 min 0% B, 22 min 90% B at 1 mL/min flow rate. The detection was carried on at λ = 220 nm.

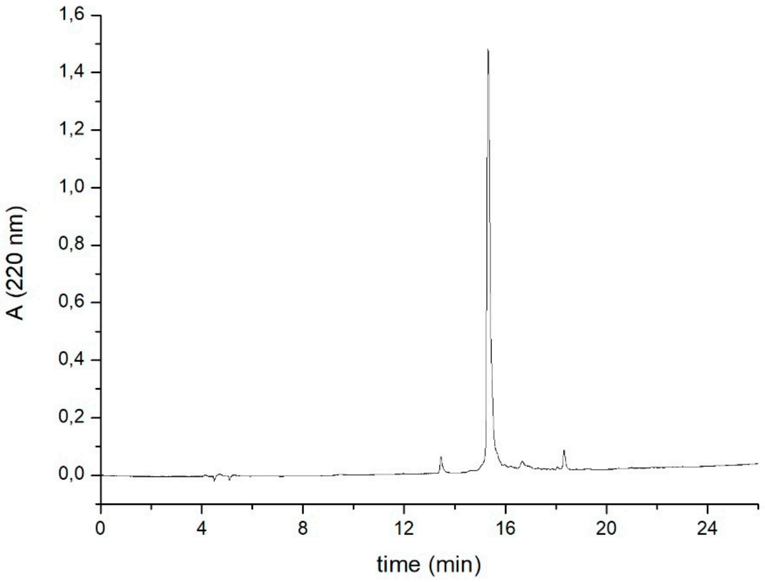

Figure S13. HPLC chromatogram of PCA-Arg$_8$-Lys(DauSuc). Retention time was obtained on Hypersil Hypurity C18 column (4.6 mm x 150 mm, 5 μm, 190 Å). The applied linear gradient elution was 0 min 0% B, 2 min 0% B, 22 min 90% B at 1 mL/min flow rate. The detection was carried on at λ = 220 nm.

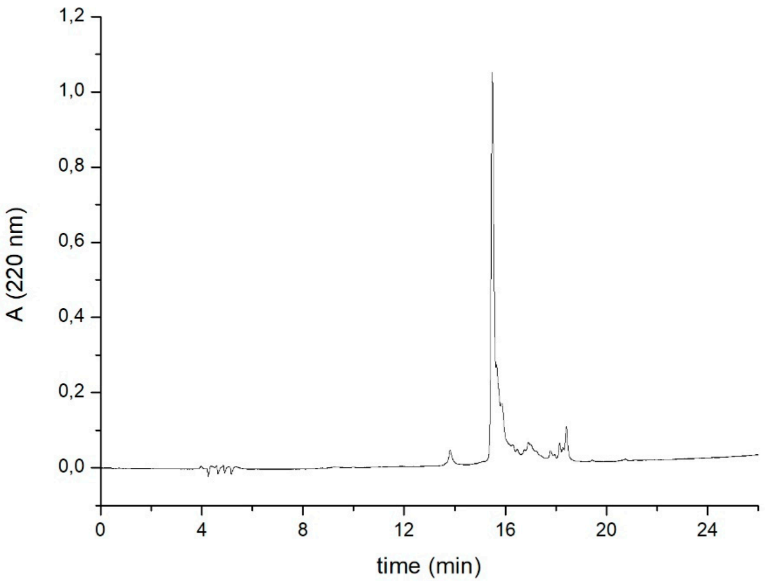

Figure S14. HPLC chromatogram of DauSuc-Arg$_8$-Lys(PCA). Retention time was obtained on Hypersil Hypurity C18 column (4.6 mm x 150 mm, 5 μm, 190 Å). The applied linear gradient elution was 0 min 0% B, 2 min 0% B, 22 min 90% B at 1 mL/min flow rate. The detection was carried on at λ = 220 nm.

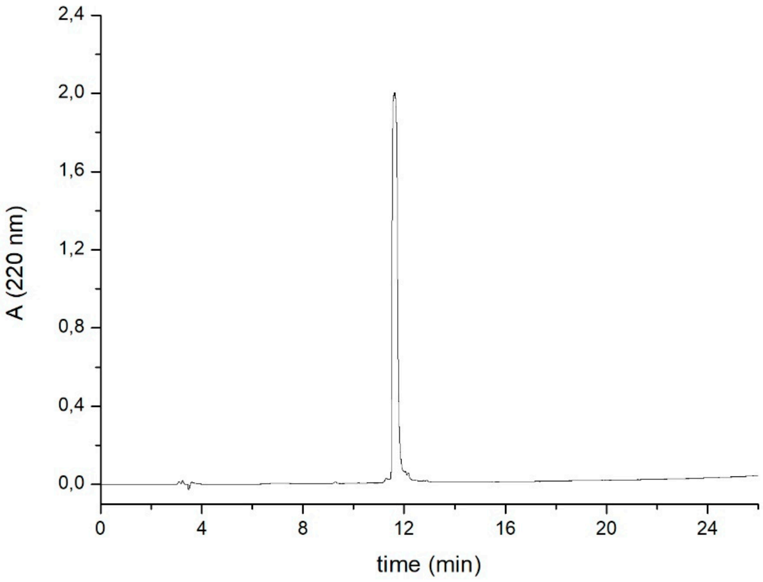

Figure S15. HPLC chromatogram of Cf-Arg8. Retention time was obtained on Hypersil Hypurity C18 column (4.6 mm x 150 mm, 5 μm, 190 Å). The applied linear gradient elution was 0 min 0% B, 2 min 0% B, 22 min 90% B at 1 mL/min flow rate. The detection was carried on at λ = 220 nm.

2.2. *Mass spectrometry*

The molecular weight of peptides and conjugates was determined with ESI-MS using Bruker Amazon SL (Germany). The samples were dissolved in a water-acetonitrile solution (50:50) with 0.1% formic acid and directly injected with a syringe pump. Parameters: capillary voltage: 4 kV, nebulizer gas: 10 psi, dry gas: 4 L/min, heated capillary temperature: 250 °C.

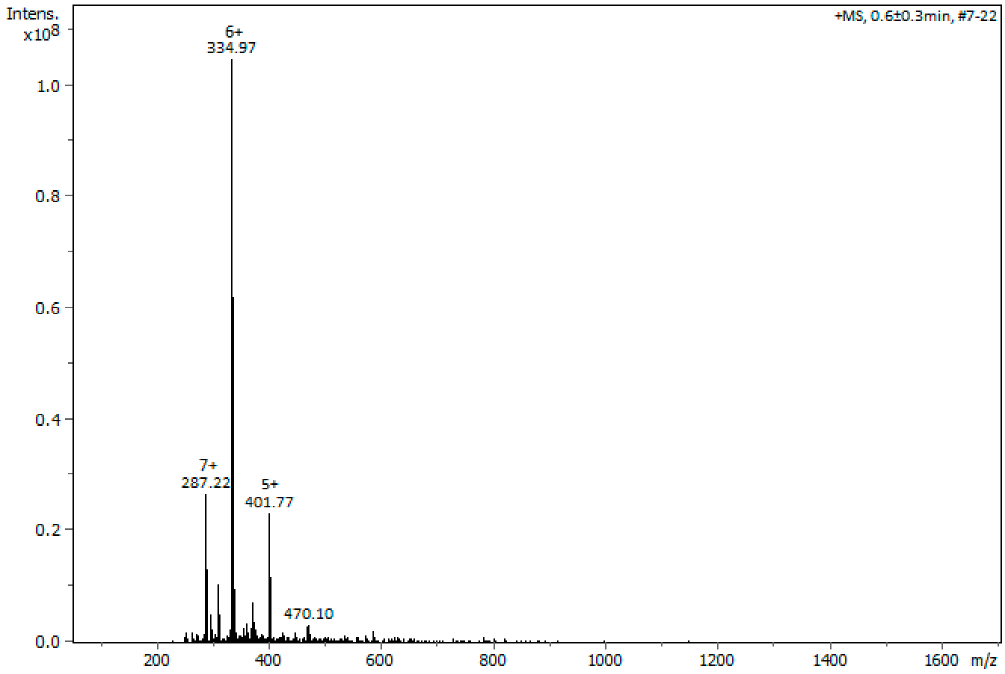

Figure S16. MS Spectrum of Dabcyl-Arg$_8$-Lys(Cf). The identity of the peptide conjugate was determined using Bruker Amazon SL (Germany). The samples are dissolved in water-acetonitrile (50:50) with 0.1% formic acid.

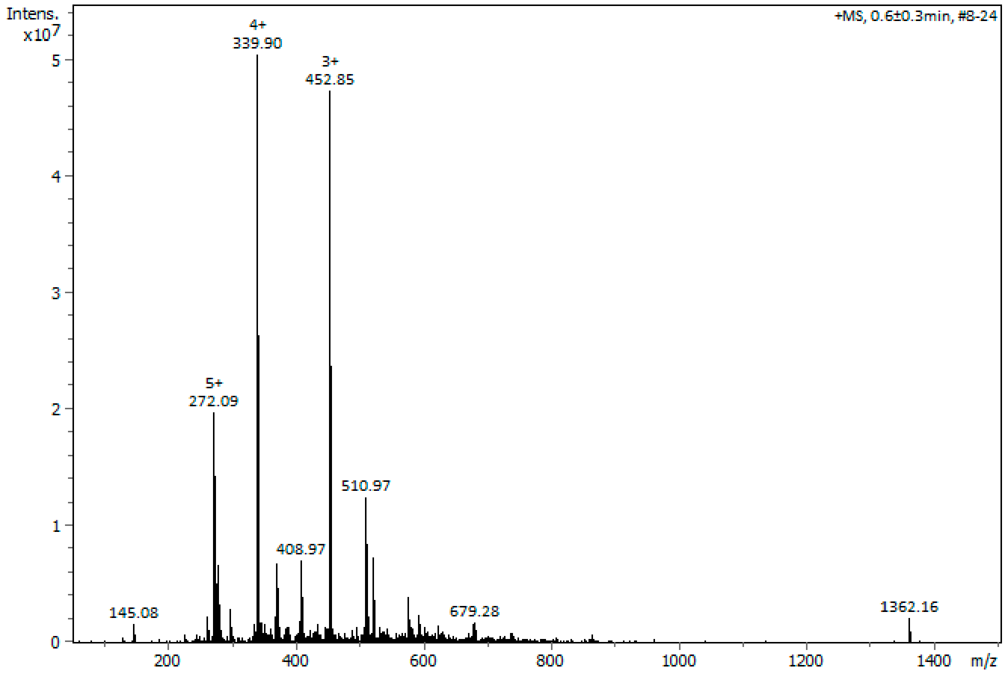

Figure S17. MS Spectrum of PCA-Arg$_4$-Lys(Cf). The identity of the peptide conjugate was determined using Bruker Amazon SL (Germany). The samples are dissolved in water-acetonitrile (50:50) with 0.1% formic acid.

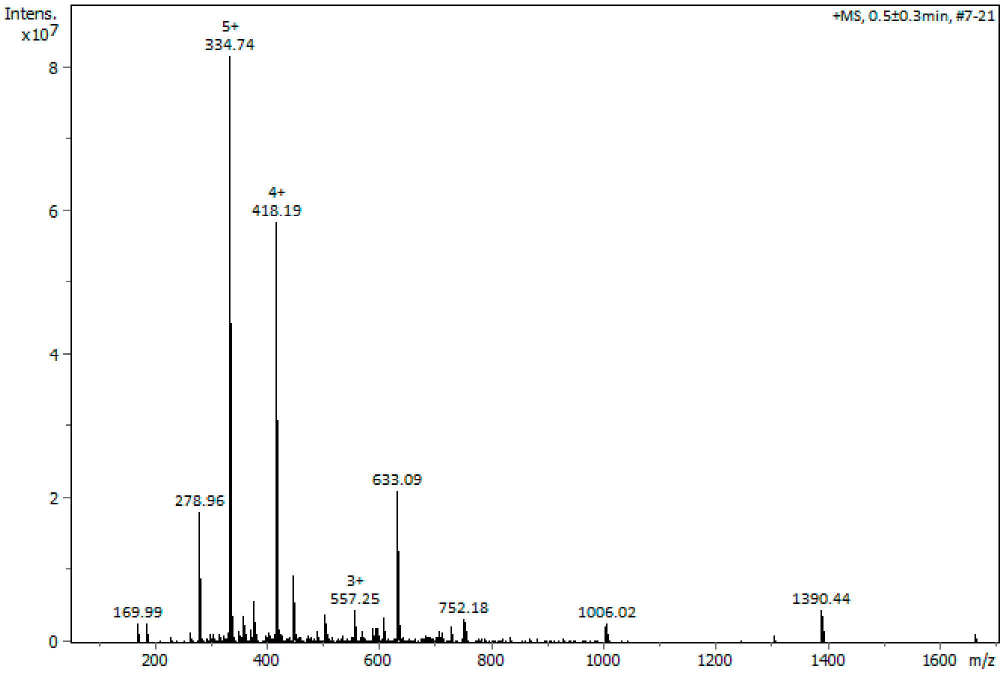

Figure S18. MS Spectrum of PCA-Arg$_6$-Lys(Cf). The identity of the peptide conjugate was determined using Bruker Amazon SL (Germany). The samples are dissolved in water-acetonitrile (50:50) with 0.1% formic acid.

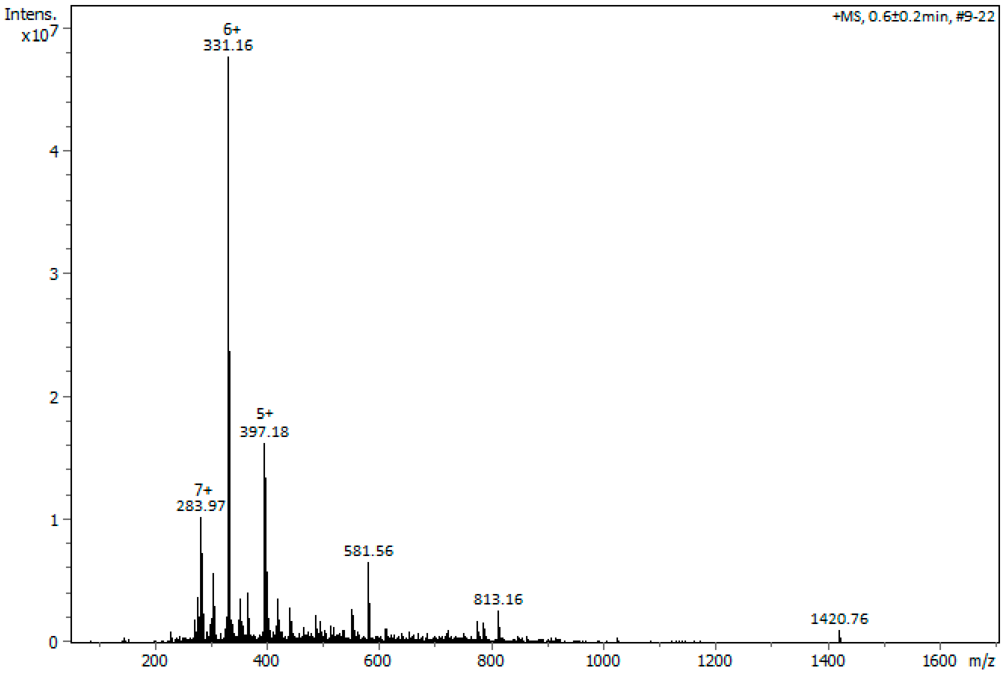

Figure S19. MS Spectrum of PCA-Arg$_8$-Lys(Cf). The identity of the peptide conjugate was determined using Bruker Amazon SL (Germany). The samples are dissolved in water-acetonitrile (50:50) with 0.1% formic acid.

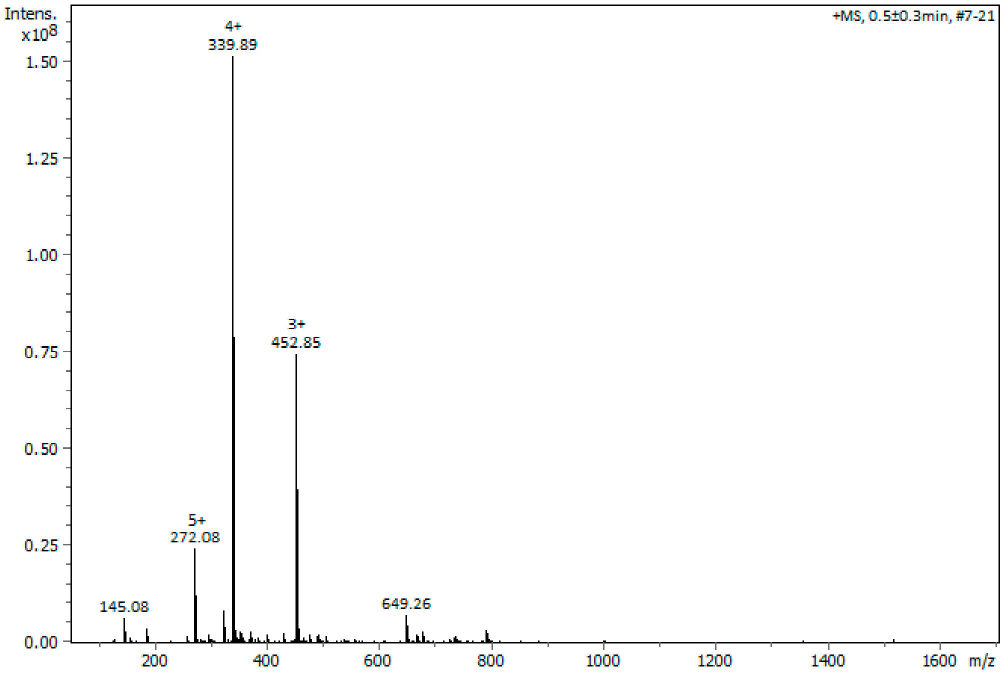

Figure S20. MS Spectrum of Cf-Arg$_4$-Lys(PCA). The identity of the peptide conjugate was determined using Bruker Amazon SL (Germany). The samples are dissolved in water-acetonitrile (50:50) with 0.1% formic acid.

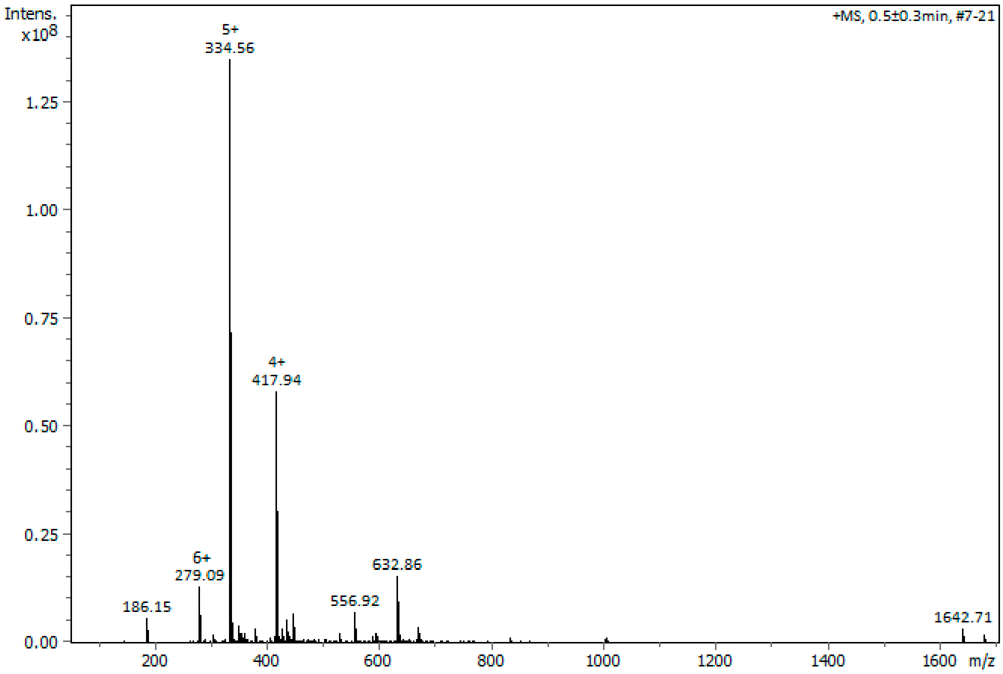

Figure S21. MS Spectrum of Cf-Arg$_6$-Lys(PCA). The identity of the peptide conjugate was determined using Bruker Amazon SL (Germany). The samples are dissolved in water-acetonitrile (50:50) with 0.1% formic acid.

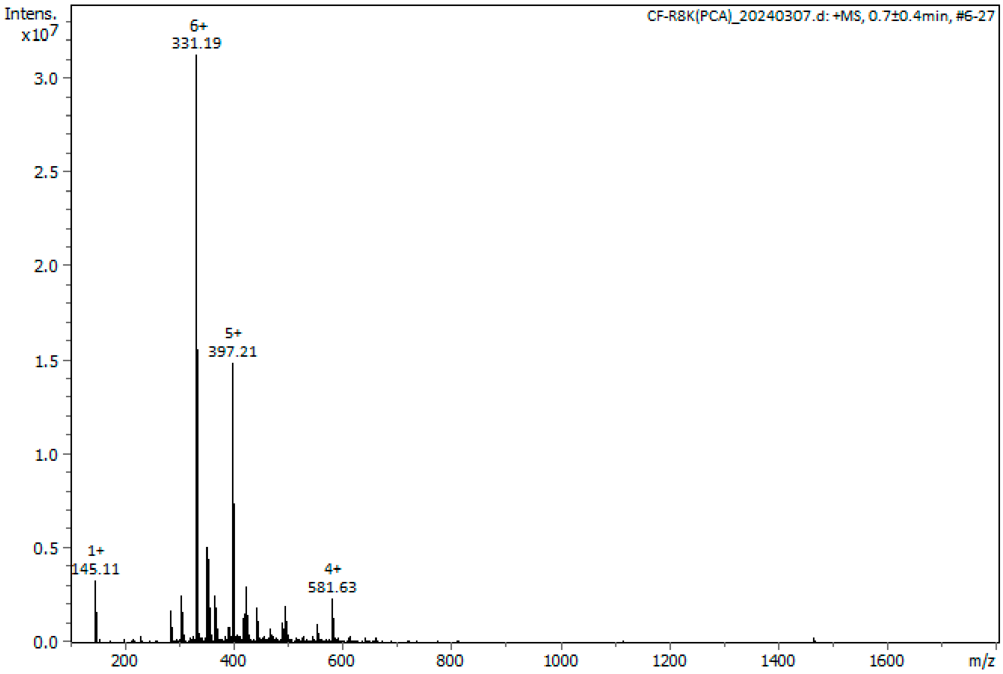

Figure S22. MS Spectrum of Cf-Arg$_8$-Lys(PCA). The identity of the peptide conjugate was determined using Bruker Amazon SL (Germany). The samples are dissolved in water-acetonitrile (50:50) with 0.1% formic acid.

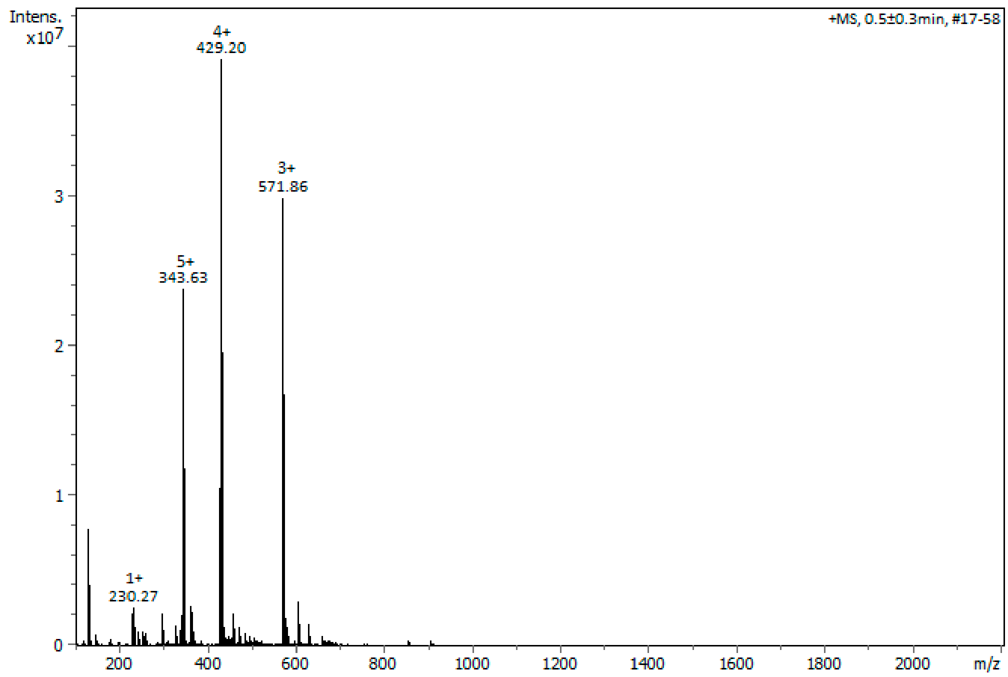

Figure S23. MS Spectrum of (PCA)$_2$-Lys-Arg$_4$-Lys(Cf). The identity of the peptide conjugate was determined using Bruker Amazon SL (Germany). The samples are dissolved in water-acetonitrile (50:50) with 0.1% formic acid.

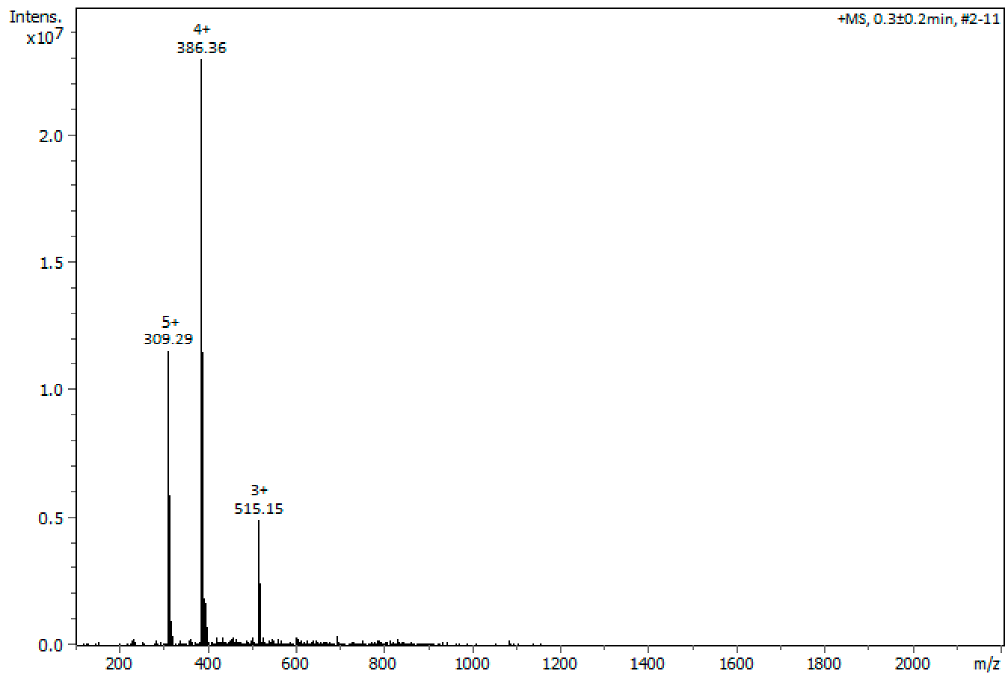

Figure S24. MS Spectrum of PCA-Trp-Arg$_4$-Lys(Cf). The identity of the peptide conjugate was determined using Bruker Amazon SL (Germany). The samples are dissolved in water-acetonitrile (50:50) with 0.1% formic acid.

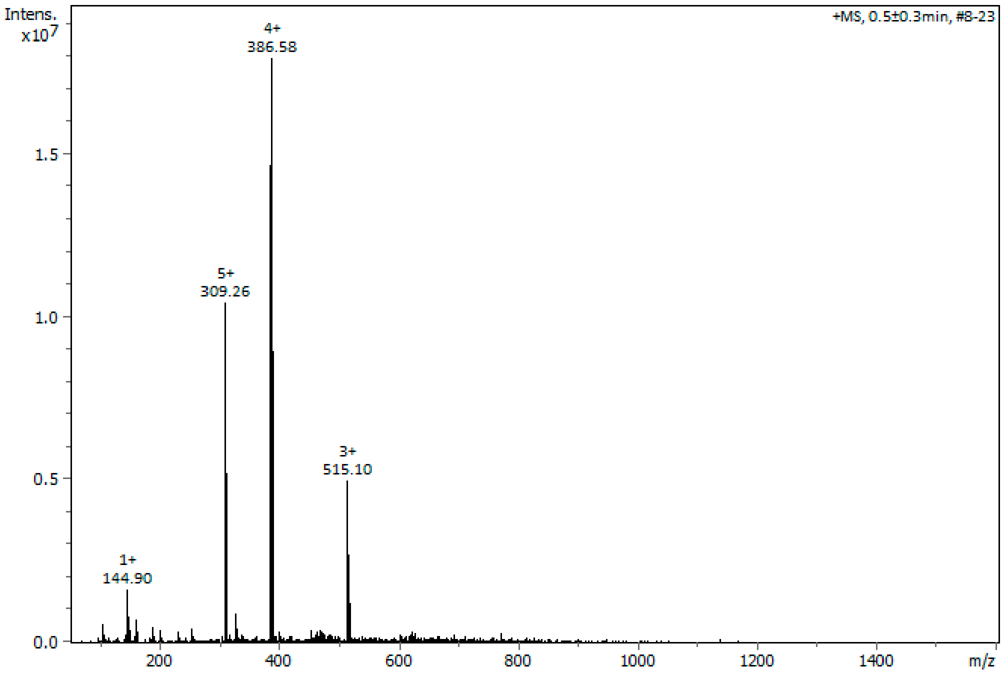

Figure S25. MS Spectrum of PCA-Arg$_2$-Trp-Arg$_2$-Lys(Cf). The identity of the peptide conjugate was determined using Bruker Amazon SL (Germany). The samples are dissolved in water-acetonitrile (50:50) with 0.1% formic acid.

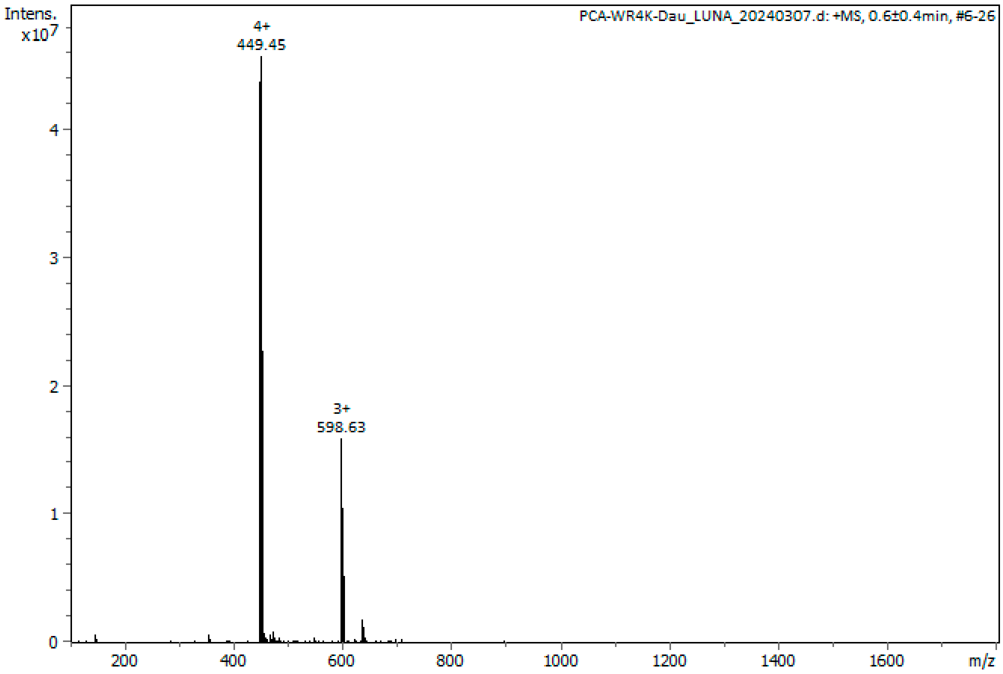

Figure S26. MS Spectrum of PCA-Trp-Arg$_4$-Lys(DauSuc). The identity of the peptide conjugate was determined using Bruker Amazon SL (Germany). The samples are dissolved in water-acetonitrile (50:50) with 0.1% formic acid.

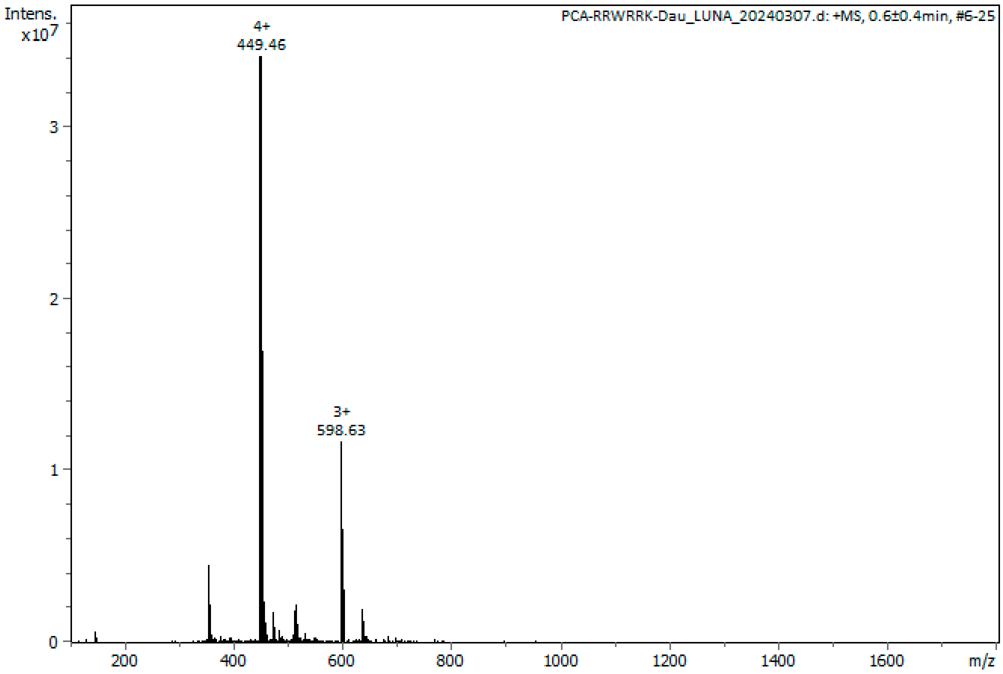

Figure S27. MS Spectrum of PCA-Arg2-Trp-Arg2-Lys(DauSuc). The identity of the peptide conjugate was determined using Bruker Amazon SL (Germany). The samples are dissolved in water-acetonitrile (50:50) with 0.1% formic acid.

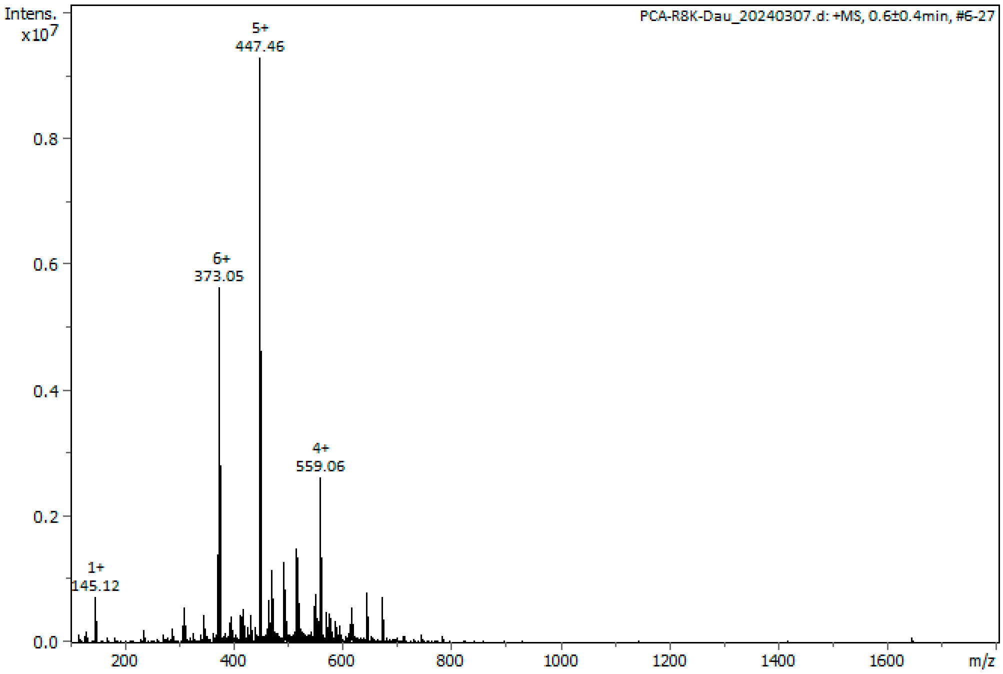

Figure S28. MS Spectrum of PCA-Arg8-Lys(DauSuc). The identity of the peptide conjugate was determined using Bruker Amazon SL (Germany). The samples are dissolved in water-acetonitrile (50:50) with 0.1% formic acid.

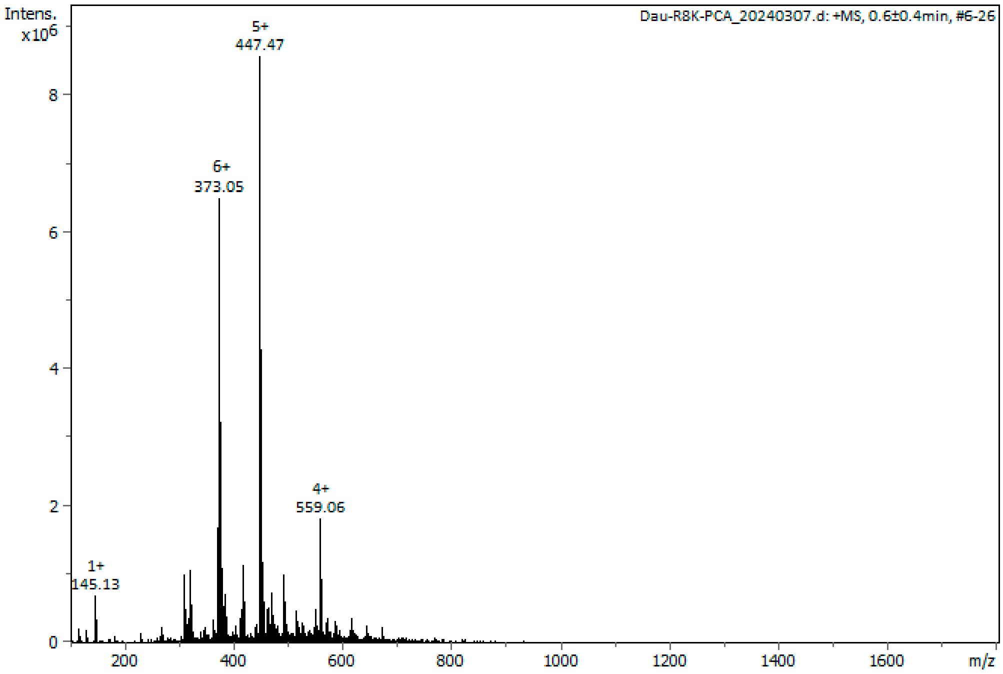

Figure S29. MS Spectrum of DauSuc-Arg$_8$-Lys(PCA). The identity of the peptide conjugate was determined using Bruker Amazon SL (Germany). The samples are dissolved in water-acetonitrile (50:50) with 0.1% formic acid.

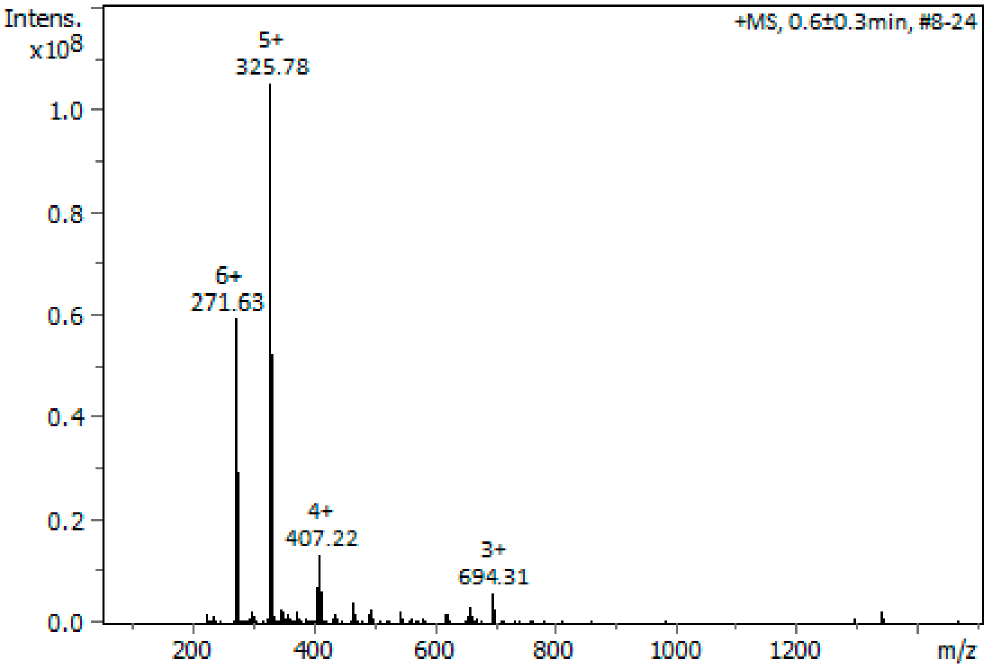

Figure S30. MS Spectrum of Cf-Arg$_8$. The identity of the peptide conjugate was determined using Bruker Amazon SL (Germany). The samples are dissolved in water-acetonitrile (50:50) with 0.1% formic acid.

3. Confocal microscopy to assess intracellular distribution

We have visualized the intracellular localization of the labeled peptides using confocal laser scanning microscopy at the 5 μM peptide concentration (based on the flow cytometry profile), and

MDA-MB 231 cells were incubated for 90 min. Representative images are presented (blue: Hoechst, nuclear dye; green: Cf-labelled peptide signal; red: Lysotracker Deep Red). Fixed MDA MB 231 cells were studied, and the treatment, staining, and fixation process were carefully optimized to assess comparable greyscale values corresponding to green (intensity of Cf) and red (intensity of LysoTracker Deep Red dye) signals.

According to the fluorescent signals, the peptides accumulated in the cytosol, partially co-localized with the lysosomal staining, and showed a different pattern.

Cf-Arg$_8$ mainly accumulated in lysosomal compartments (based on lysosomal staining). Co-localization with the lysosomal staining can indicate vesicular transport in the peptide uptake. For the Cf-Arg$_8$, the Cf signal only partially co-localizes with the cytoplasmic area. Low co-localization with the nuclei staining can be observed.

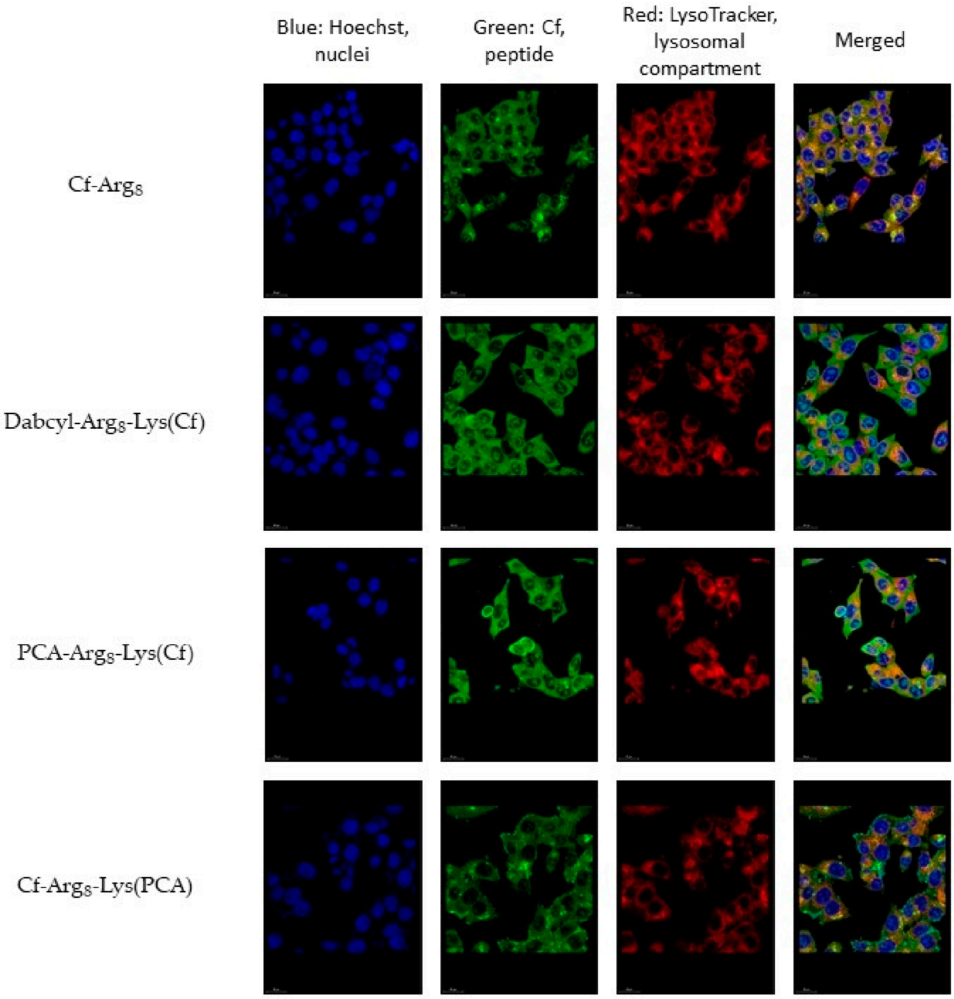

Figure S31. Intracellular localization of peptides visualized by confocal microscopy. MDA-MB-231 cells were treated with the peptide conjugates (5 μM) for 90 min.

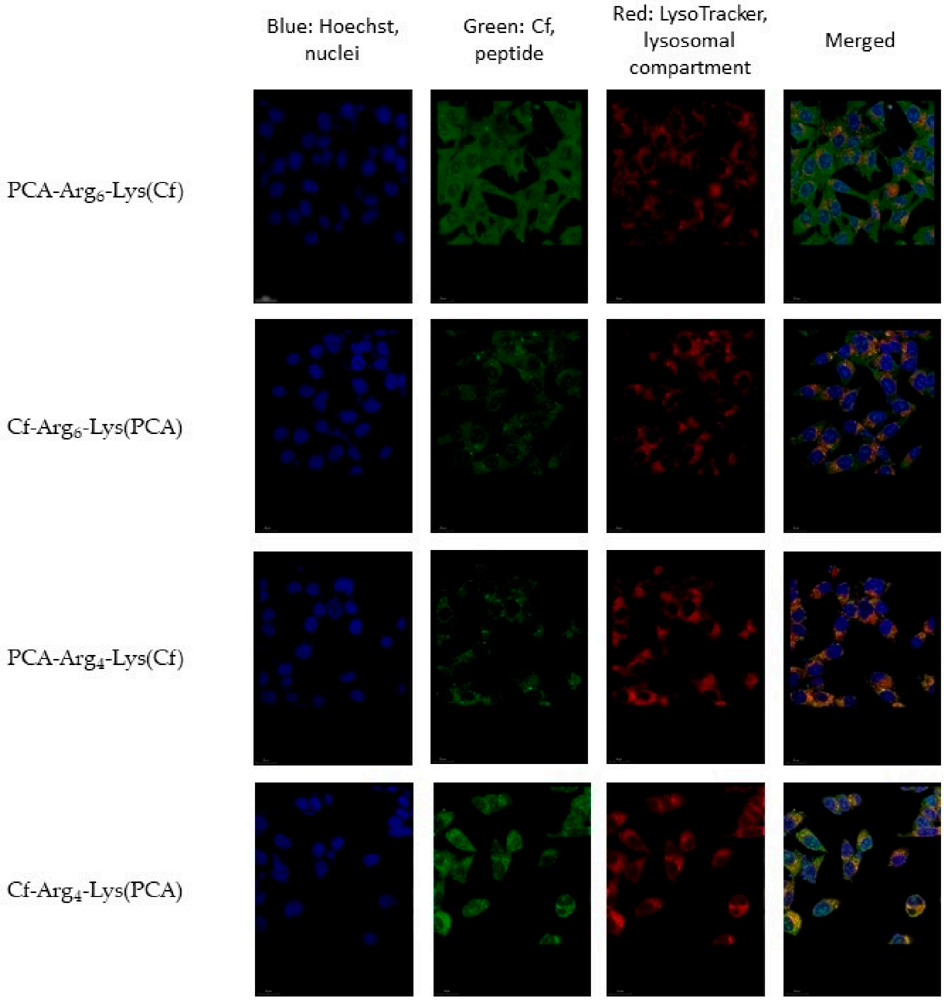

Figure S32. Intracellular localization of peptides visualized by confocal microscopy. MDA-MB-231 cells were treated with the peptide conjugates (5 μM) for 90 min.

4. Cytotoxicity measurements of the peptide conjugates

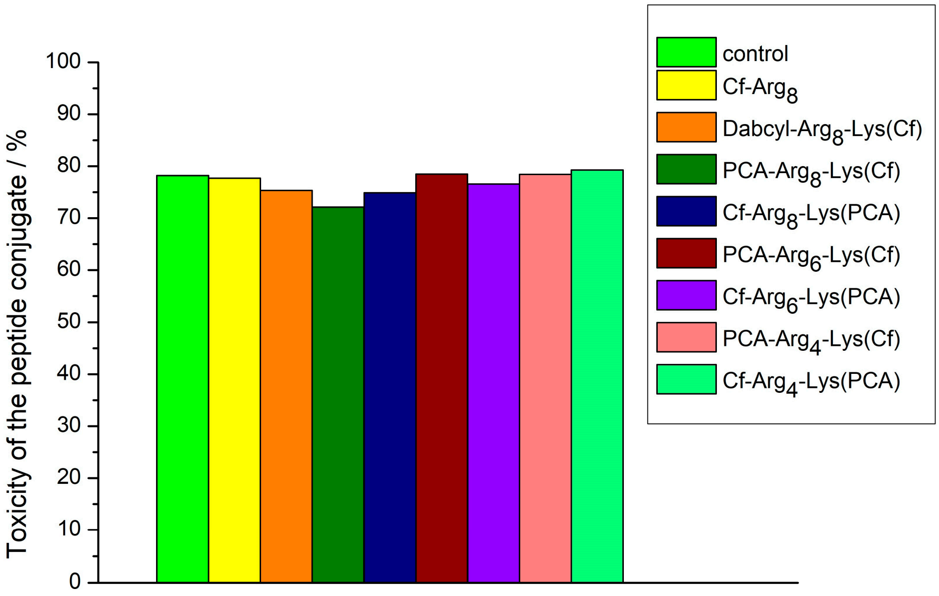

Figure S33. The cytotoxicity of the peptide conjugates measured on MDA-MB-231 cells in 5 μM.

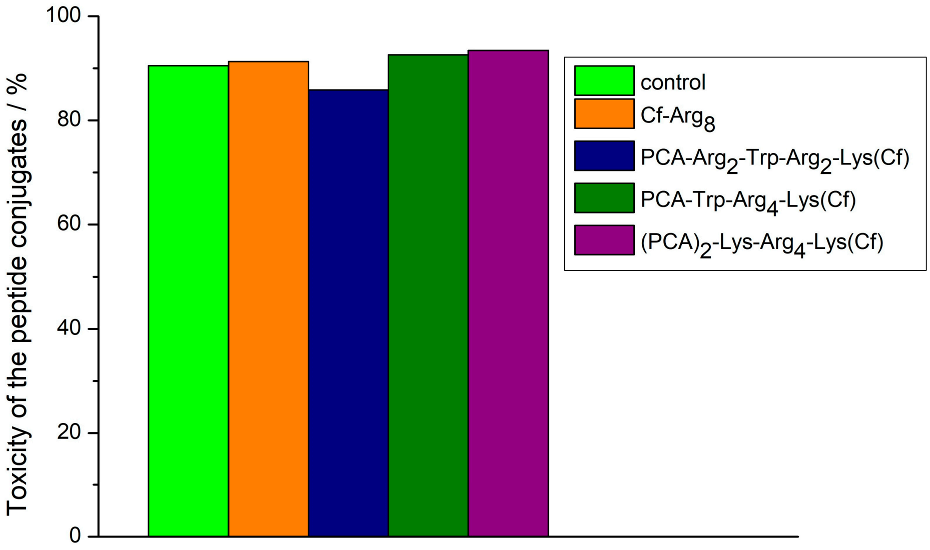

Figure S34. The cytotoxicity of the peptide conjugates measured on MDA-MB-231 cells in 10 ɥM.

5. Internalization and intracellular localization analysis based on confocal microscopic images

The Cf-labelled peptides' cellular internalization was captured by a confocal microscope. The intracellular localization was assessed using the following approaches:

Analysis of the unprocessed images with LasX software of Leica SP8. (Figure S35 – Table S1, Figure S38 - Table S4, Figure S41 - Table S7, Figure S44 - Table S10, Figure S47 - Table S13, Figure S50 - Table S16, Figure S53 - Table S19, Figure S56 - Table S22)

"In-house Python-based" image analysis. (Figure S36 – Table S2, Figure S39 - Table S5, Figure S42 - Table S8, Figure S45 - Table S11, Figure S48 - Table S14, Figure S51 - Table S17, Figure S54 - Table S20, Figure S57 - Table S23)

The unprocessed images (blue, green, red) will allow us to track the peptide uptake separately according to cellular niches (nucleus, lysosome) by color. First, we normalize each pixel intensity value [49] and then flatten the images [49] (reduce the two-dimensional array – the pixel coordinates – into a one-dimensional list of normalized intensity values) for each channel. These lists of values were used to calculate the Pearson correlations [50,51] (red-green lysosomal, blue-green nuclear).

Based on our previous work [52,53], we evaluated the co-localization using ImageJ JACoP using the processed images from the LasX software of Leica SP8. (Figure S37 – Table S3, Figure S40 - Table S6, Figure S43 - Table S9, Figure S46 - Table S12, Figure S49 - Table S15, Figure S52 - Table S18, Figure S55 - Table S21, Figure S58 - Table S24)

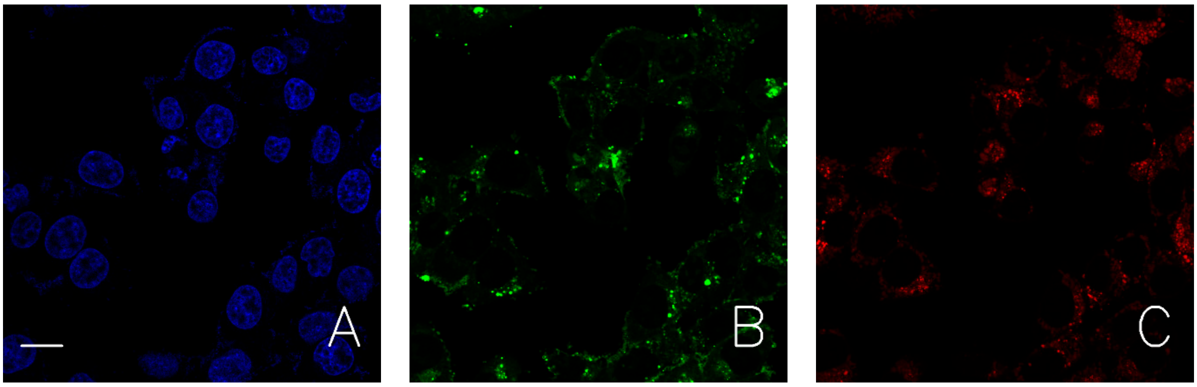

Figure S35. Internalization of peptide Cf-Arg_8-Lys(PCA) captured by confocal laser scanning microscopy using unprocessed images. Cells were incubated for 90 minutes with peptide (c=5 μM). Nuclei were stained with Hoechst 33342, blue (A); Cf labelled peptide, green (B); lysosomes were labelled with LysoTracker Deep Red, red (C). Cells were examined by Leica SP8 using an HC PL APO CS2 63x/1.40 OIL objective with the hybrid detector. The scale bar represents 20 μm.

Table S1. Co-localization profile of peptide Cf-Arg_8-Lys(PCA). We applied Leica LasX software using unprocessed images for image analysis and co-localization study. (A) red-green channels; (B) blue-green channels.

**(A)**	**Colocalization**	
Pearson's Correlation	0,3946	
Overlap Coefficient	0,5233	
Colocalization Rate	52,77%	
Colocalization Area [m^2]	6,83E-10	
Area Image [m^2]	2,10E-08	
Area Foreground [m^2]	1,29E-09	
Area Background [m^2]	1,97E-08	
	Channel Green	Channel Red
Mean Intensity Image	11,36	8,35
Mean Intensity Colocalization	47,76	58,38
Intensity Sum Image	1,19E+07	8756391
Intensity Sum Colocalization	1629439	1991979
**(B)**	**Colocalization**	
Pearson's Correlation	0,109	
Overlap Coefficient	0,307	
Colocalization Rate	11,74%	
Colocalization Area [m^2]	2,53E-10	
Area Image [m^2]	2,10E-08	
Area Foreground [m^2]	2,16E-09	
Area Background [m^2]	1,88E-08	
	Channel Green	Channel Blue
Mean Intensity Image	18,53	11,36
Mean Intensity Colocalization	69,19	71,75
Intensity Sum Image	1,94E+07	1,19E+07
Intensity Sum Colocalization	876073	908451

Table S2. Co-localization analysis using single-channel confocal "tif" images (unprocessed images from Leica LasX software).

**Pearson Correlation** (Red-Green)[a]	**Pearson Correlation** (Blue-Green)[b]	L_{coeff} (Red)[c]	L_{coeff} (Blue)[d]	**Average Intensity** (Green in Red Uptake)[e]	**Average Intensity** (Green in Blue Uptake)[f]	**Average Intensity** (Green)[g]
0.3916	0.1087	0.9973	0.9917	11.38	11.81	1.56

The „in-house Python-based" method calculates the "Pearson Correlations," the Lcoeff (Red, Blue), and the average intensity values of green channel intensities. We created categories by filtering the channels based on varying criteria. We also count the sum of the number of pixels of each category. We developed metrics based on channel overlap and used these metrics to calculate the L_{coeff} (Red, Blue).

[a]Value calculated by a Python module [50] from the flattened red and green channels. Relative value (-1-1) that reflects the linear correlation of two sets of the intensity values of the red and green channel pixels

[b]Value calculated by a Python module [50] from the flattened red and green channels. Relative value (-1-1) that reflects the linear correlation of two sets of the intensity values of the blue and green channel pixels

[c]Co-localization value calculated from red and green channel pixel counts employing the developed equation

[d]Co-localization value calculated from blue and green channel pixel counts employing the developed equation

[e]Calculated average intensity of peptides co-localized with lysosomes

[f]Calculated average intensity of peptides co-localized with nuclei

[g]Calculated average intensity of peptides in cell cytosol/cytoplasm

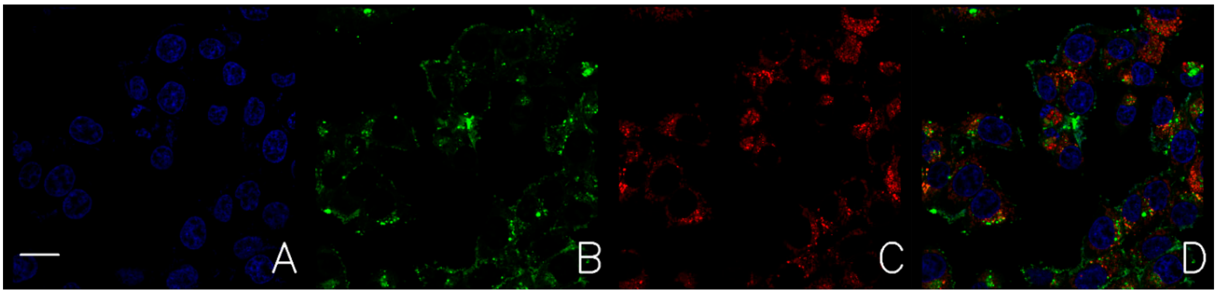

Figure S36. Internalization of peptide Cf-Arg_8-Lys(PCA) captured by confocal laser scanning microscopy using processed images. Cells were incubated for 90 minutes with peptide (c=5 μM). Nuclei were stained with Hoechst 33342, blue (A); Cf labelled peptide, green (B); lysosomes were labelled with LysoTracker Deep Red, red (C); merged channels (D). Cells were examined by Leica SP8 confocal microscope with adaptive lightning mode using an HC PL APO CS2 63x/1.40 OIL objective with the hybrid detector. The scale bar represents 20 μm.

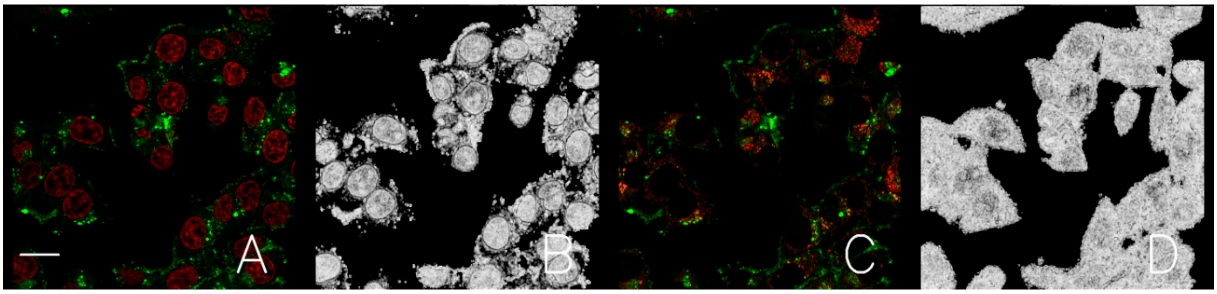

Figure S37. Costes' mask and spatial Pearson images were retrieved from **Figure S36.** with ImageJ using JACoP. [54,55] Costes' Mask: ImageJ JACoP performs Costes' automatic threshold analysis. The created mask highlights the co-localized regions of the compared channels. [54,55] Costes's Mask, blue and green channels compared from **Figure S36.** partD (A); Spatial Pearson map, blue and green channels compared from **Figure S36.** partD (B); Costes' Mask, red and green channels compared from **Figure S36.** partD (C); Spatial Pearson map, red and green channels compared from **Figure S36.** partD (D).

Table S3. Based on our previous studies [52,53], we have also evaluated the co-localization using ImageJ JACoP. [54,55]

		Red-Green	Blue-Green
**Pearson Coefficient**[a]		0.372	0.06
**Manders Coefficients**[b]	M1	0.996	0.96
(original)	M2	0.948	0.627

[a] This is a linear equation that uses linear regression to calculate the relationship between the intensities of two images. 1 value stands for complete correlation, and -1 stands for complete negative correlation, while zero stands for no correlation at all

[b]Manders' overlap coefficient is based on Pearson but takes out the average intensities. The values range from 0 to 1. M1 and M2 are relative values between the sum intensity of the channel, whereas the other channel has a value above zero compared to the total sum intensity of the channel. [54,55]

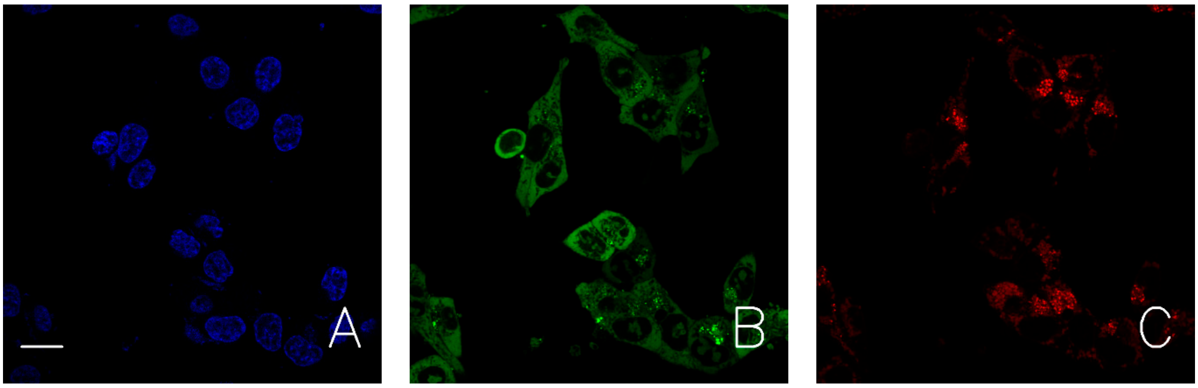

Figure S38. Internalization of peptide PCA-Arg_8-Lys(Cf) captured by confocal laser scanning microscopy using unprocessed images. Cells were incubated for 90 minutes with peptide (c=5 μM). Nuclei were stained with Hoechst 33342, blue (A); Cf labelled peptide, green (B); lysosomes were labelled with LysoTracker Deep Red, red (C). Cells were examined by Leica SP8 using an HC PL APO CS2 63x/1.40 OIL objective with the hybrid detector. The scale bar represents 20 μm.

Table S4. Co-localization profile of peptide PCA-Arg_8-Lys(Cf). We applied Leica LasX software using unprocessed images for image analysis and co-localization study. (A) red-green channels; (B) blue-green channels.

**(A)**	**Colocalization**	
Pearson's Correlation	0,4516	
Overlap Coefficient	0,5278	
Colocalization Rate	43,41%	
Colocalization Area [m^2]	1,09E-09	
Area Image [m^2]	2,10E-08	
Area Foreground [m^2]	2,50E-09	
Area Background [m^2]	1,85E-08	
	Channel Green	Channel Red
Mean Intensity Image	12,04	5,73
Mean Intensity Colocalization	52,3	49,02
Intensity Sum Image	1,26E+07	6009235
Intensity Sum Colocalization	2838686	2660744

(B)	Colocalization	
Pearson's Correlation	0,2298	
Overlap Coefficient	0,3416	
Colocalization Rate	21,88%	
Colocalization Area [m^2]	3,75E-10	
Area Image [m^2]	2,10E-08	
Area Foreground [m^2]	1,72E-09	
Area Background [m^2]	1,93E-08	
	Channel Green	Channel Blue
Mean Intensity Image	9,47	12,04
Mean Intensity Colocalization	81	58,92
Intensity Sum Image	9933976	1,26E+07
Intensity Sum Colocalization	1519633	1105404

Table S5. Co-localization analysis using single-channel confocal "tif" images (unprocessed images from Leica LasX software).

**Pearson Correlation** (Red-Green)[a]	**Pearson Correlation** (Blue-Green)[b]	L_{coeff} (Red)[c]	L_{coeff} (Blue)[d]	**Average Intensity** (Green in Red Uptake)[e]	**Average Intensity** (Green in Blue Uptake)[f]	**Average Intensity** (Green)[g]
0.4493	0.2344	0.9930	0.9669	22.76	21.35	1.33

The „in-house Python-based" method calculates the "Pearson Correlations," the Lcoeff (Red, Blue), and the average intensity values of green channel intensities. We created categories by filtering the channels based on varying criteria. We also count the sum of the number of pixels of each category. We developed metrics based on channel overlap and used these metrics to calculate the L_{coeff} (Red, Blue).

[a]Value calculated by a Python module [50] from the flattened red and green channels. Relative value (-1-1) that reflects the linear correlation of two sets of the intensity values of the red and green channel pixels

[b]Value calculated by a Python module [50] from the flattened red and green channels. Relative value (-1-1) that reflects the linear correlation of two sets of the intensity values of the blue and green channel pixels

[c]Co-localization value calculated from red and green channel pixel counts employing the developed equation

[d]Co-localization value calculated from blue and green channel pixel counts employing the developed equation

[e]Calculated average intensity of peptides co-localized with lysosomes

[f]Calculated average intensity of peptides co-localized with nuclei

[g]Calculated average intensity of peptides in cell cytosol/cytoplasm

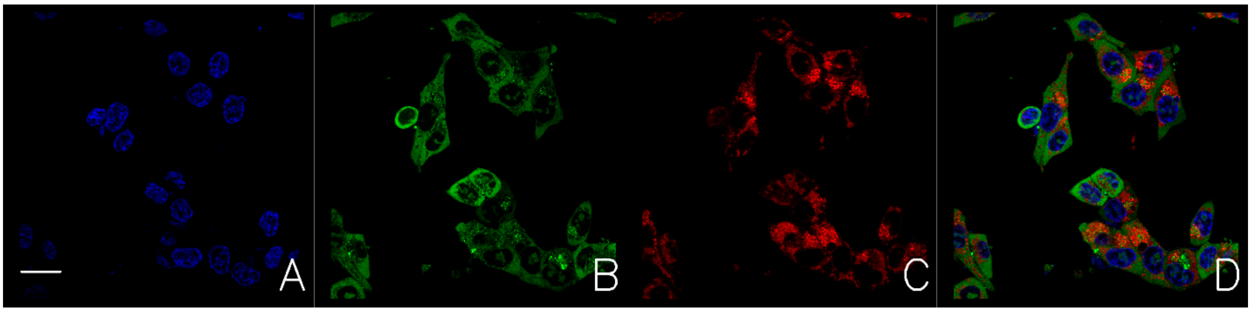

Figure S39. Internalization of peptide PCA-Arg_8-Lys(Cf) captured by confocal laser scanning microscopy using processed images. Cells were incubated for 90 minutes with peptide (c=5 μM). Nuclei were stained with Hoechst 33342, blue (A); Cf labelled peptide, green (B); lysosomes were labelled with LysoTracker Deep Red, red (C); merged channels (D). Cells were examined by Leica SP8 confocal microscope with adaptive lightning mode using an HC PL APO CS2 63x/1.40 OIL objective with the hybrid detector. The scale bar represents 20 μm.

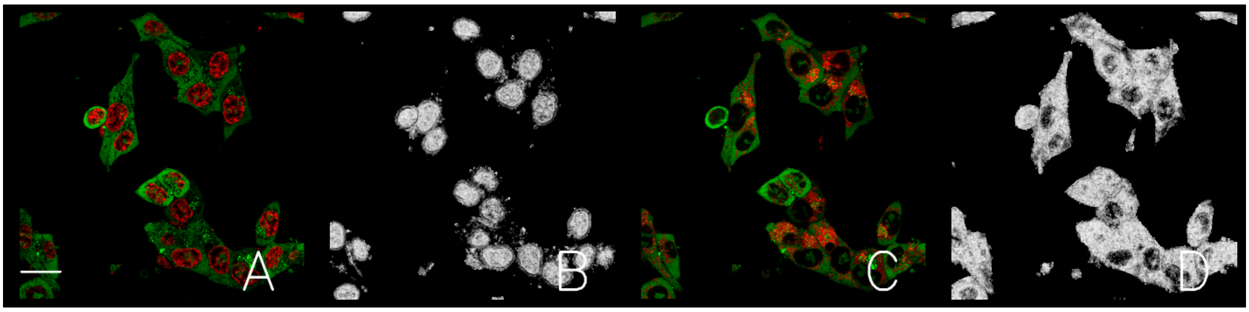

Figure S40. Costes' mask and spatial Pearson images were retrieved from **Figure S39.** with ImageJ using JACoP. [54,55] Costes' Mask: ImageJ JACoP performs Costes' automatic threshold analysis. The created mask highlights the co-localized regions of the compared channels. [54,55] Costes's Mask, blue and green channels compared from **Figure S39.** partD (A); Spatial Pearson map, blue and green channels compared from **Figure S39.** partD (B); Costes' Mask, red and green channels compared from **Figure S39.** partD (C); Spatial Pearson map, red and green channels compared from **Figure S39.** partD (D).

Table S6. Based on our previous studies, [52,53] we have also evaluated the co-localization using ImageJ JACoP. [54,55]

		**Red-Green**	**Blue-Green**
**Pearson Coefficient**[a]		0.487	0.151
**Manders Coefficients**[b]	**M1**	0. 997	0.976
(original)	**M2**	0. 799	0.33

[a] This is a linear equation that uses linear regression to calculate the relationship between the intensities of two images. 1 value stands for complete correlation, and -1 stands for complete negative correlation, while zero stands for no correlation at all

[b]Manders' overlap coefficient is based on Pearson but takes out the average intensities. The values range from 0 to 1. M1 and M2 are relative values between the sum intensity of the channel, whereas the other channel has a value above zero compared to the total sum intensity of the channel. [54,55]

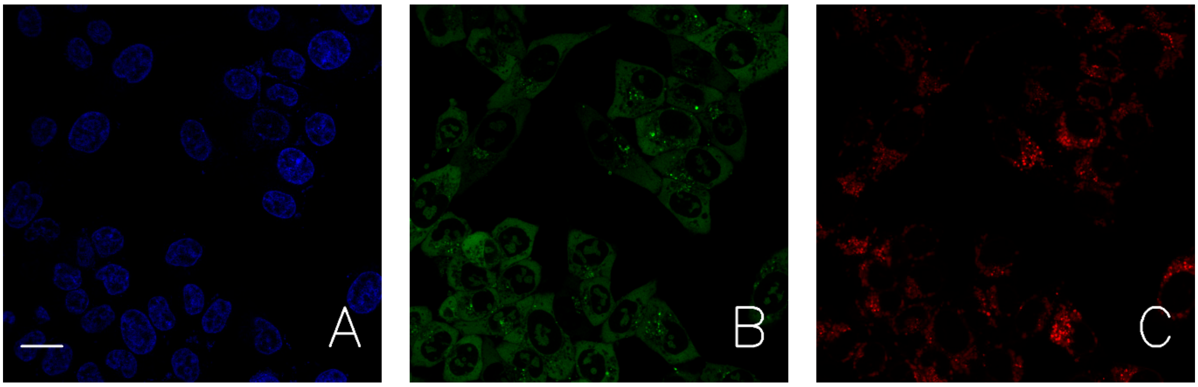

Figure S41. Internalization of peptide Dabcyl-Arg$_8$-Lys(Cf) by confocal laser scanning microscopy using unprocessed images. Cells were incubated for 90 minutes with peptide (c=5 μM). Nuclei were stained with Hoechst 33342, blue (A); Cf labelled peptide, green (B); lysosomes were labelled with LysoTracker Deep Red, red (C). Cells were examined by Leica SP8 using a HC PL APO CS2 63x/1.40 OIL objective with hybrid detector. The scale bar represents 20 μm.

Table S7. Co-localization profile of peptide Dabcyl-Arg$_8$-Lys(Cf). For image analysis and co-localization study we applied Leica LasX software using unprocessed images. (A) red-green channels; (B) blue-green channels.

**(A)**	**Colocalization**	
Pearson's Correlation	0,4321	
Overlap Coefficient	0,5939	
Colocalization Rate	52,82%	
Colocalization Area [m²]	1,18E-09	
Area Image [m²]	2,10E-08	
Area Foreground [m²]	2,24E-09	
Area Background [m²]	1,87E-08	
	Channel Green	Channel Red
Mean Intensity Image	15,34	8,87
Mean Intensity Colocalization	43,74	48,05
Intensity Sum Image	1,61E+07	9303519
Intensity Sum Colocalization	2582325	2837118
**(B)**	**Colocalization**	
Pearson's Correlation	0,1293	
Overlap Coefficient	0,3948	
Colocalization Rate	19,76%	
Colocalization Area [m²]	2,70E-10	
Area Image [m²]	2,10E-08	
Area Foreground [m²]	1,37E-09	
Area Background [m²]	1,96E-08	
	Channel Green	Channel Blue
Mean Intensity Image	15,72	15,34
Mean Intensity Colocalization	79,2	44,25
Intensity Sum Image	1,65E+07	1,61E+07
Intensity Sum Colocalization	1067338	596377

Table S8. Co-localization analysis using single-channel confocal "tif" images (unprocessed images from Leica LasX software).

**Pearson Correlation** (Red-Green)[a]	**Pearson Correlation** (Blue-Green)[b]	**L$_{coeff}$** (Red)[c]	**L$_{coeff}$** (Blue)[d]	**Average Intensity** (Green in Red Uptake)[e]	**Average Intensity** (Green in Blue Uptake)[f]	**Average Intensity** (Green)[g]
0.4323	0.1342	0.9925	0.9859	15.28	14.8	1.68

The „in-house Python-based" method calculates the "Pearson Correlations," the Lcoeff (Red, Blue), and the average intensity values of green channel intensities. We created categories by filtering the channels based on varying criteria. We also count the sum of the number of pixels of each category. We developed metrics based on channel overlap and used these metrics to calculate the L$_{coeff}$ (Red, Blue).

[a]Value calculated by a Python module [50] from the flattened red and green channels. Relative value (-1-1) that reflects the linear correlation of two sets of the intensity values of the red and green channel pixels

[b]Value calculated by a Python module [50] from the flattened red and green channels. Relative value (-1-1) that reflects the linear correlation of two sets of the intensity values of the blue and green channel pixels

[c]Co-localization value calculated from red and green channel pixel counts employing the developed equation

[d]Co-localization value calculated from blue and green channel pixel counts employing the developed equation
[e]Calculated average intensity of peptides co-localized with lysosomes
[f]Calculated average intensity of peptides co-localized with nuclei
[g]Calculated average intensity of peptides in cell cytosol/cytoplasm

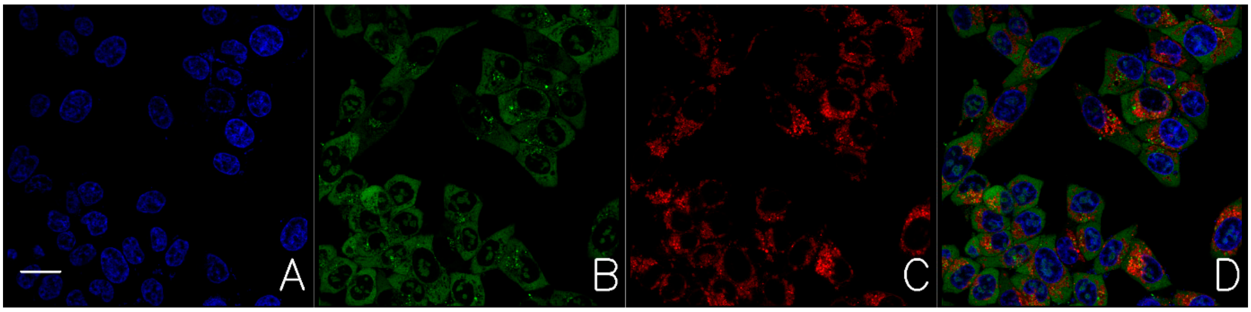

Figure S42. Internalization of peptide Dabcyl-Arg$_8$-Lys(Cf) captured by confocal laser scanning microscopy using processed images. Cells were incubated for 90 minutes with peptide (c=5 μM). Nuclei were stained with Hoechst 33342, blue (A); Cf labelled peptide, green (B); lysosomes were labelled with LysoTracker Deep Red, red (C); merged channels (D). Cells were examined by Leica SP8 confocal microscope with adaptive lightning mode using an HC PL APO CS2 63x/1.40 OIL objective with the hybrid detector. The scale bar represents 20 μm.

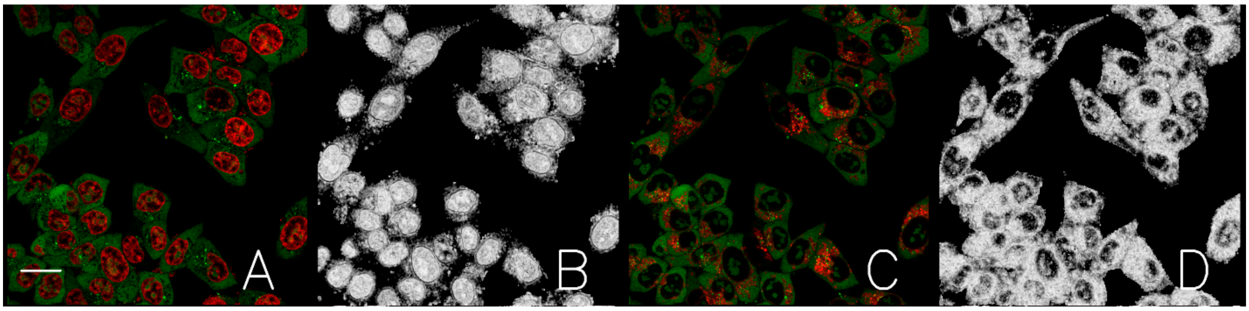

Figure S43. Costes' mask and spatial Pearson images were retrieved from **Figure S42.** with ImageJ using JACoP. [54,55] Costes' Mask: ImageJ JACoP performs Costes' automatic threshold analysis. The created mask highlights the co-localized regions of the compared channels. [54,55] Costes's Mask, blue and green channels compared from **Figure S42.** partD (A); Spatial Pearson map, blue and green channels compared from **Figure S42.** partD (B); Costes' Mask, red and green channels compared from **Figure S42.** partD (C); Spatial Pearson map, red and green channels compared from **Figure S42.** partD (D).

Table S9. Based on our previous studies, [52,53] we have also evaluated the co-localization using ImageJ JACoP. [54,55]

		Red-Green	Blue-Green
**Pearson Coefficient**[a]		0.392	0.048
**Manders Coefficients**[b]	**M1**	0.993	0.953
(original)	**M2**	0.725	0.52

[a] This is a linear equation that uses linear regression to calculate the relationship between the intensities of two images. 1 value stands for complete correlation, and -1 stands for complete negative correlation, while zero stands for no correlation at all
[b]Manders' overlap coefficient is based on Pearson but takes out the average intensities. The values range from 0 to 1. M1 and M2 are relative values between the sum intensity of the channel, whereas the other channel has a value above zero compared to the total sum intensity of the channel. [54,55]

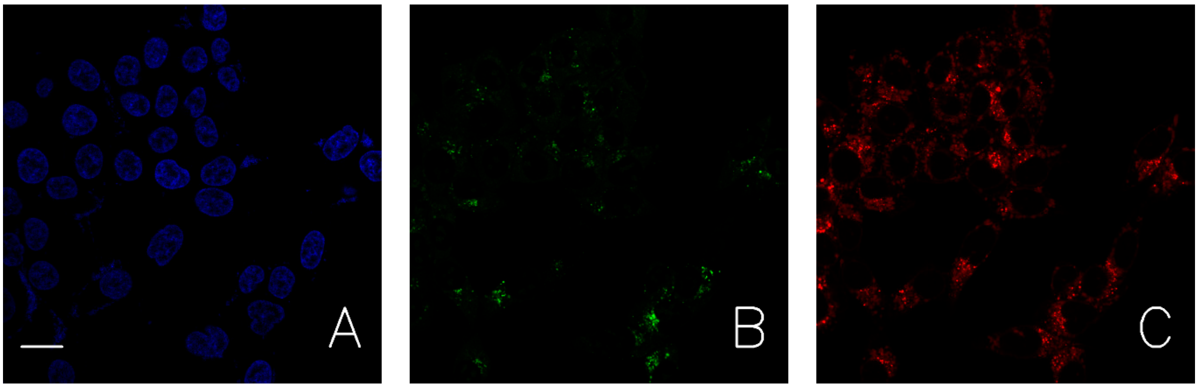

Figure S44. Internalization of peptide Cf-Arg$_8$ captured by confocal laser scanning microscopy using unprocessed images. Cells were incubated for 90 minutes with peptide (c=5 μM). Nuclei were stained with Hoechst 33342, blue (A); Cf labelled peptide, green (B); lysosomes were labelled with LysoTracker Deep Red, red (C). Cells were examined by Leica SP8 using an HC PL APO CS2 63x/1.40 OIL objective with the hybrid detector. The scale bar represents 20 μm.

Table S10. Co-localization profile of peptide Cf-Arg$_8$. We applied Leica LasX software using unprocessed images for image analysis and co-localization study. (A) red-green channels; (B) blue-green channels.

**(A)**	**Colocalization**	
Pearson's Correlation	0,6449	
Overlap Coefficient	0,7178	
Colocalization Rate	51,74%	
Colocalization Area [m²]	4,95E-10	
Area Image [m²]	2,10E-08	
Area Foreground [m²]	9,56E-10	
Area Background [m²]	2,00E-08	
	Channel Green	Channel Red
Mean Intensity Image	4,94	9,82
Mean Intensity Colocalization	39,03	65,73
Intensity Sum Image	5179251	1,03E+07
Intensity Sum Colocalization	964746	1624600
**(B)**	**Colocalization**	
Pearson's Correlation	0,0987	
Overlap Coefficient	0,2748	
Colocalization Rate	0,68%	
Colocalization Area [m²]	7,61E-12	
Area Image [m²]	2,10E-08	
Area Foreground [m²]	1,13E-09	
Area Background [m²]	1,99E-08	

	Channel Green	Channel Blue
Mean Intensity Image	12,82	4,94
Mean Intensity Colocalization	69,98	56,04
Intensity Sum Image	1,34E+07	5179251
Intensity Sum Colocalization	26591	21294

Table S11. Co-localization analysis using single-channel confocal "tif" images (unprocessed images from Leica LasX software).

**Pearson Correlation** (Red-Green)[a]	**Pearson Correlation** (Blue-Green)[b]	**L_{coeff}** (Red)[c]	**L_{coeff}** (Blue)[d]	**Average Intensity** (Green in Red Uptake)[e]	**Average Intensity** (Green in Blue Uptake)[f]	**Average Intensity** (Green)[g]
0.6410	0.1001	0.9676	0.9642	5.91	5.32	1.12

The „in-house Python-based" method calculates the "Pearson Correlations," the Lcoeff (Red, Blue), and the average intensity values of green channel intensities. We created categories by filtering the channels based on varying criteria. We also count the sum of the number of pixels of each category. We developed metrics based on channel overlap and used these metrics to calculate the L_{coeff} (Red, Blue).

[a]Value calculated by a Python module [50] from the flattened red and green channels. Relative value (-1-1) that reflects the linear correlation of two sets of the intensity values of the red and green channel pixels

[b]Value calculated by a Python module [50] from the flattened red and green channels. Relative value (-1-1) that reflects the linear correlation of two sets of the intensity values of the blue and green channel pixels

[c]Co-localization value calculated from red and green channel pixel counts employing the developed equation

[d]Co-localization value calculated from blue and green channel pixel counts employing the developed equation

[e]Calculated average intensity of peptides co-localized with lysosomes

[f]Calculated average intensity of peptides co-localized with nuclei

[g]Calculated average intensity of peptides in cell cytosol/cytoplasm

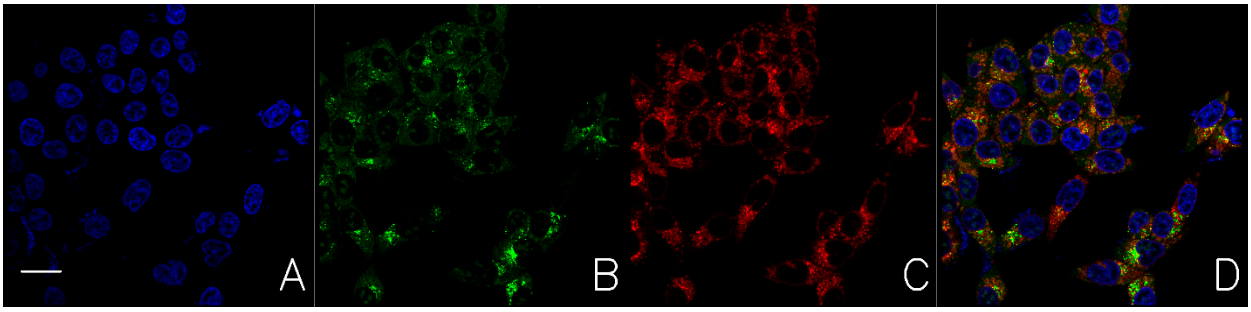

Figure S45. Internalization of peptide Cf-Arg$_8$ captured by confocal laser scanning microscopy using processed images. Cells were incubated for 90 minutes with peptide (c=5 μM). Nuclei were stained with Hoechst 33342, blue (A); Cf labelled peptide, green (B); lysosomes were labelled with LysoTracker Deep Red, red (C); merged channels (D). Cells were examined by Leica SP8 confocal microscope with adaptive lightning mode using an HC PL APO CS2 63x/1.40 OIL objective with the hybrid detector. The scale bar represents 20 μm.

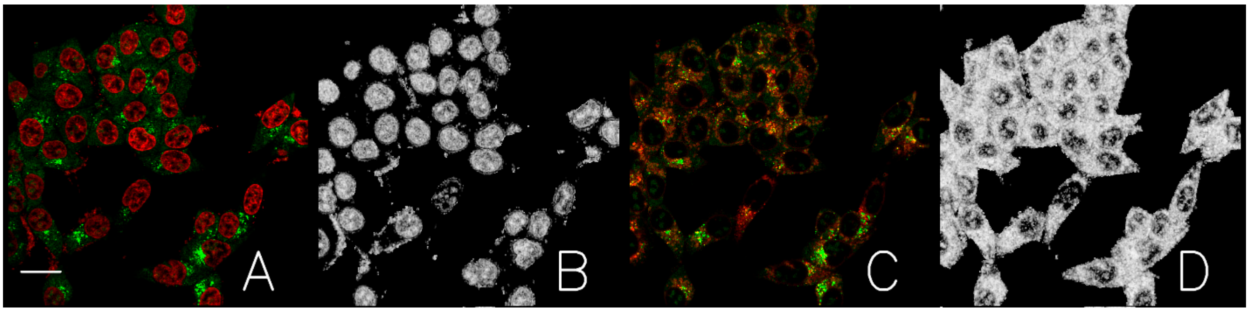

Figure S46. Costes' mask and spatial Pearson images were retrieved from **Figure S45.** with ImageJ using JACoP. [54,55] Costes' Mask: ImageJ JACoP performs Costes' automatic threshold analysis. The created mask highlights the co-localized regions of the compared channels. [54,55] Costes's Mask, blue and green channels compared from **Figure S45.** partD (A); Spatial Pearson map, blue and green channels compared from **Figure S45.** partD (B); Costes' Mask, red and green channels compared from **Figure S45.** partD (C); Spatial Pearson map, red and green channels compared from **Figure S45.** partD (D).

Table S12. Based on our previous studies, [52,53] we have also evaluated the co-localization using ImageJ JACoP. [54,55]

		Red-Green	Blue-Green
**Pearson Coefficient**[a]		0.688	0.037
**Manders Coefficients**[b]	**M1**	0.941	0.787
(original)	**M2**	0.957	0.315

[a] This is a linear equation that uses linear regression to calculate the relationship between the intensities of two images. 1 value stands for complete correlation, and -1 stands for complete negative correlation, while zero stands for no correlation at all

[b]Manders' overlap coefficient is based on Pearson but takes out the average intensities. The values range from 0 to 1. M1 and M2 are relative values between the sum intensity of the channel, whereas the other channel has a value above zero compared to the total sum intensity of the channel. [54,55]

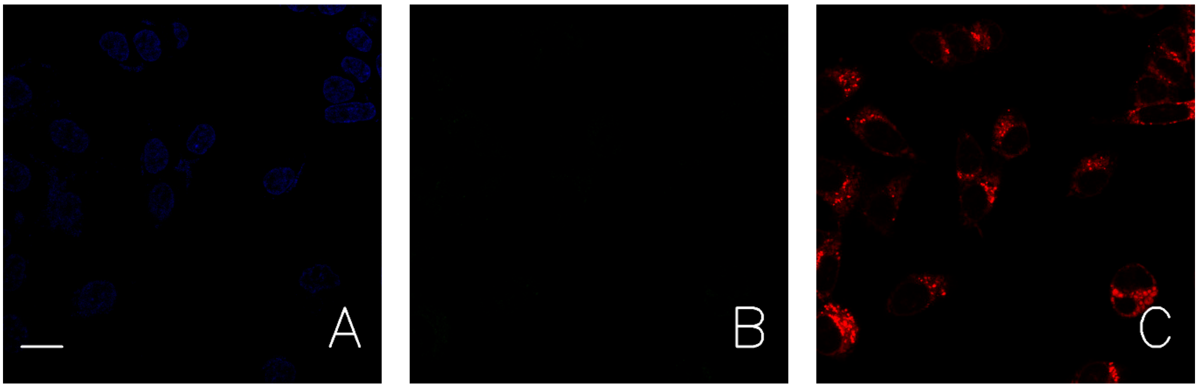

Figure S47. Internalization of peptide Cf-Arg$_4$-Lys(PCA) captured by confocal laser scanning microscopy using unprocessed images. Cells were incubated for 90 minutes with peptide (c=5 µM). Nuclei were stained with Hoechst 33342, blue (A); Cf labelled peptide, green (B); lysosomes were labelled with LysoTracker Deep Red, red (C). Cells were examined by Leica SP8 using an HC PL APO CS2 63x/1.40 OIL objective with the hybrid detector. The scale bar represents 20 µm.

Table S13. Co-localization profile of peptide Cf-Arg$_4$-Lys(PCA). We applied Leica LasX software using unprocessed images for image analysis and co-localization study. (A) red-green channels; (B) blue-green channels.

**(A)**	**Colocalization**	
Pearson's Correlation	0,5646	
Overlap Coefficient	0,6246	
Colocalization Rate	1,63%	
Colocalization Area [m^2]	1,17E-11	
Area Image [m^2]	2,10E-08	
Area Foreground [m^2]	7,17E-10	
Area Background [m^2]	2,03E-08	
	Channel Green	Channel Red
Mean Intensity Image	1,6	7,63
Mean Intensity Colocalization	19,32	57,13
Intensity Sum Image	1682313	7997587
Intensity Sum Colocalization	11265	33306
**(B)**	**Colocalization**	
Pearson's Correlation	0,2841	
Overlap Coefficient	0,3845	
Colocalization Rate	0,01%	
Colocalization Area [m^2]	2,00E-14	
Area Image [m^2]	2,10E-08	
Area Foreground [m^2]	1,42E-10	

Area Background [m²]	2,08E-08	
	Channel Green	Channel Blue
Mean Intensity Image	4,3	1,6
Mean Intensity Colocalization	88	27
Intensity Sum Image	4504794	1682313
Intensity Sum Colocalization	88	27

Table S14. Co-localization analysis using single-channel confocal "tif" images (unprocessed images from Leica LasX software).

**Pearson Correlation** (Red-Green)[a]	**Pearson Correlation** (Blue-Green)[b]	L_{coeff} (Red)[c]	L_{coeff} (Blue)[d]	**Average Intensity** (Green in Red Uptake)[e]	**Average Intensity** (Green in Blue Uptake)[f]	**Average Intensity** (Green)[g]
0.5653	0.2858	0.7361	0.8183	3.07	3.03	1.21

The „in-house Python-based" method calculates the "Pearson Correlations," the Lcoeff (Red, Blue), and the average intensity values of green channel intensities. We created categories by filtering the channels based on varying criteria. We also count the sum of the number of pixels of each category. We developed metrics based on channel overlap and used these metrics to calculate the L_{coeff} (Red, Blue).

[a]Value calculated by a Python module [50] from the flattened red and green channels. Relative value (-1-1) that reflects the linear correlation of two sets of the intensity values of the red and green channel pixels

[b]Value calculated by a Python module [50] from the flattened red and green channels. Relative value (-1-1) that reflects the linear correlation of two sets of the intensity values of the blue and green channel pixels

[c]Co-localization value calculated from red and green channel pixel counts employing the developed equation

[d]Co-localization value calculated from blue and green channel pixel counts employing the developed equation

[e]Calculated average intensity of peptides co-localized with lysosomes

[f]Calculated average intensity of peptides co-localized with nuclei

[g]Calculated average intensity of peptides in cell cytosol/cytoplasm

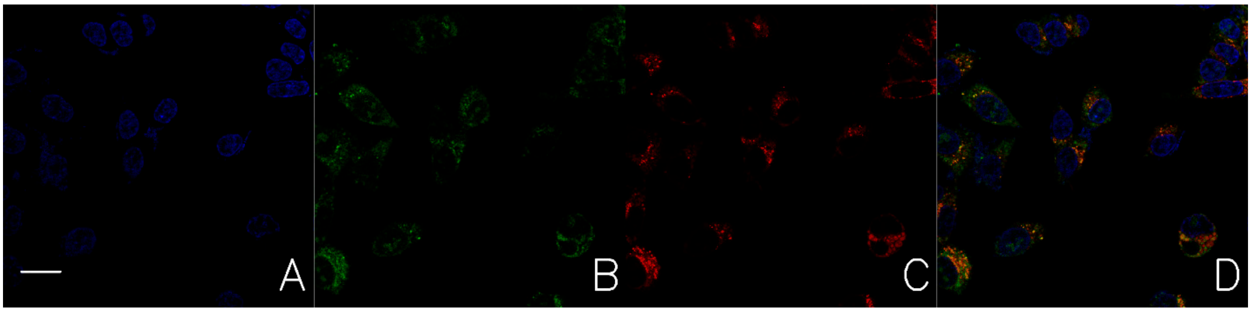

Figure S48. Internalization of peptide Cf-Arg$_4$-Lys(PCA) captured by confocal laser scanning microscopy using processed images. Cells were incubated for 90 minutes with peptide (c=5 μM). Nuclei were stained with Hoechst 33342, blue (A); Cf labelled peptide, green (B); lysosomes were labelled with LysoTracker Deep Red, red (C); merged channels (D). Cells were examined by Leica SP8 confocal microscope with adaptive lightning mode using an HC PL APO CS2 63x/1.40 OIL objective with the hybrid detector. The scale bar represents 20 μm.

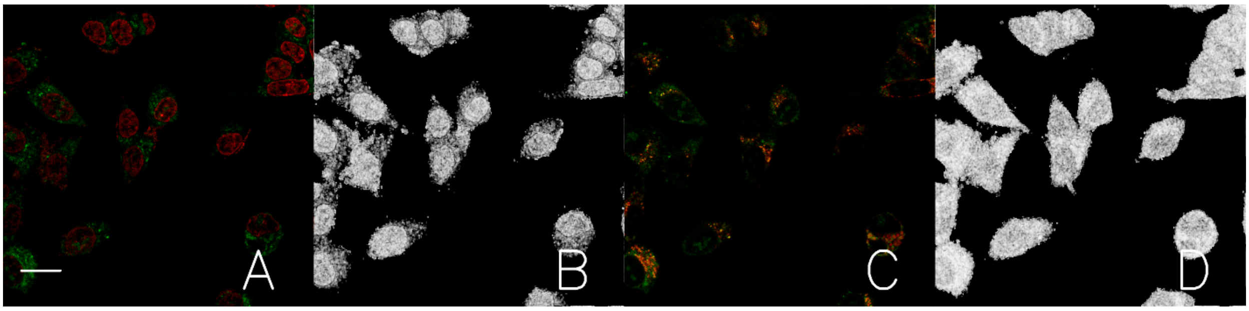

Figure S49. Costes' mask and spatial Pearson images were retrieved from **Figure S48.** with ImageJ using JACoP. [54,55] Costes' Mask: ImageJ JACoP performs Costes' automatic threshold analysis. The created mask highlights the co-localized regions of the compared channels. [54,55] Costes's Mask, blue and green channels compared from **Figure S48.** partD (A); Spatial Pearson map, blue and green channels compared from **Figure S48.** partD (B); Costes' Mask, red and green channels compared from **Figure S48.** partD (C); Spatial Pearson map, red and green channels compared from **Figure S48.** partD (D).

Table S15. Based on our previous studies, [52,53] we have also evaluated the co-localization using ImageJ JACoP. [54,55]

		Red-Green	Blue-Green
**Pearson Coefficient**[a]		0.742	0.225
**Manders Coefficients**[b]	**M1**	0.992	0.988
(original)	**M2**	0.993	0.686

[a] This is a linear equation that uses linear regression to calculate the relationship between the intensities of two images. 1 value stands for complete correlation, and -1 stands for complete negative correlation, while zero stands for no correlation at all

[b]Manders' overlap coefficient is based on Pearson but takes out the average intensities. The values range from 0 to 1. M1 and M2 are relative values between the sum intensity of the channel, whereas the other channel has a value above zero compared to the total sum intensity of the channel. [54,55]

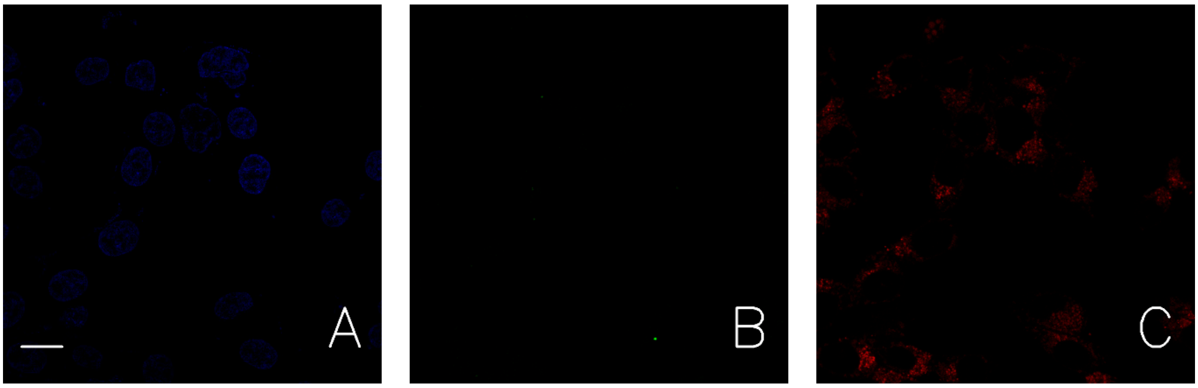

Figure S50. Internalization of peptide PCA-Arg$_4$-Lys(Cf) captured by confocal laser scanning microscopy using unprocessed images. Cells were incubated for 90 minutes with peptide (c=5 μM). Nuclei were stained with Hoechst 33342, blue (A); Cf labelled peptide, green (B); lysosomes were labelled with LysoTracker Deep Red, red (C). Cells were examined by Leica SP8 using an HC PL APO CS2 63x/1.40 OIL objective with the hybrid detector. The scale bar represents 20 μm.

Table S16. Co-localization profile of peptide PCA-Arg$_4$-Lys(Cf). We applied Leica LasX software using unprocessed images for image analysis and co-localization study. (A) red-green channels; (B) blue-green channels.

**(A)**	**Colocalization**	
Pearson's Correlation	0,4379	
Overlap Coefficient	0,5187	
Colocalization Rate	1,86%	
Colocalization Area [m^2]	4,54E-12	
Area Image [m^2]	2,10E-08	
Area Foreground [m^2]	2,45E-10	
Area Background [m^2]	2,07E-08	
	Channel Green	Channel Red
Mean Intensity Image	1,01	4,6
Mean Intensity Colocalization	31,3	52,24
Intensity Sum Image	1059220	4826361
Intensity Sum Colocalization	7106	11859
**(B)**	**Colocalization**	
Pearson's Correlation	0,1015	
Overlap Coefficient	0,2124	
Colocalization Rate	0,02%	
Colocalization Area [m^2]	2,00E-14	
Area Image [m^2]	2,10E-08	
Area Foreground [m^2]	1,30E-10	
Area Background [m^2]	2,09E-08	

	Channel Green	Channel Blue
Mean Intensity Image	4,69	1,01
Mean Intensity Colocalization	54	57
Intensity Sum Image	4913295	1059220
Intensity Sum Colocalization	54	57

Table S17. Co-localization analysis using single-channel confocal "tif" images (unprocessed images from Leica LasX software).

**Pearson Correlation** (Red-Green)[a]	**Pearson Correlation** (Blue-Green)[b]	**L_{coeff}** (Red)[c]	**L_{coeff}** (Blue)[d]	**Average Intensity** (Green in Red Uptake)[e]	**Average Intensity** (Green in Blue Uptake)[f]	**Average Intensity** (Green)[g]
0.4363	0.1000	0.5756	0.5426	2.24	2.03	1.19

The „in-house Python-based" method calculates the "Pearson Correlations," the Lcoeff (Red, Blue), and the average intensity values of green channel intensities. We created categories by filtering the channels based on varying criteria. We also count the sum of the number of pixels of each category. We developed metrics based on channel overlap and used these metrics to calculate the L_{coeff} (Red, Blue).

[a]Value calculated by a Python module [50] from the flattened red and green channels. Relative value (-1-1) that reflects the linear correlation of two sets of the intensity values of the red and green channel pixels

[b]Value calculated by a Python module [50] from the flattened red and green channels. Relative value (-1-1) that reflects the linear correlation of two sets of the intensity values of the blue and green channel pixels

[c]Co-localization value calculated from red and green channel pixel counts employing the developed equation

[d]Co-localization value calculated from blue and green channel pixel counts employing the developed equation

[e]Calculated average intensity of peptides co-localized with lysosomes

[f]Calculated average intensity of peptides co-localized with nuclei

[g]Calculated average intensity of peptides in cell cytosol/cytoplasm

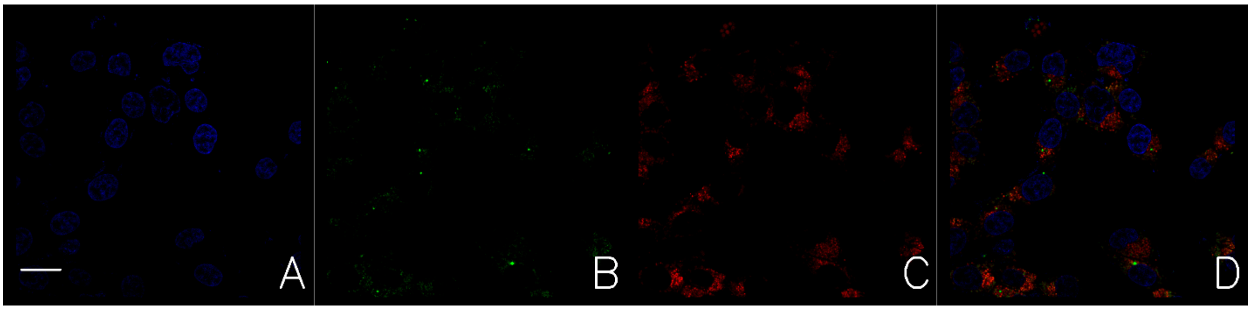

Figure S51. Internalization of peptide PCA-Arg$_4$-Lys(Cf) captured by confocal laser scanning microscopy using processed images. Cells were incubated for 90 minutes with peptide (c=5 µM). Nuclei were stained with Hoechst 33342, blue (A); Cf labelled peptide, green (B); lysosomes were labelled with LysoTracker Deep Red, red (C); merged channels (D). Cells were examined by Leica SP8 confocal microscope with adaptive lightning mode using an HC PL APO CS2 63x/1.40 OIL objective with the hybrid detector. The scale bar represents 20 µm.

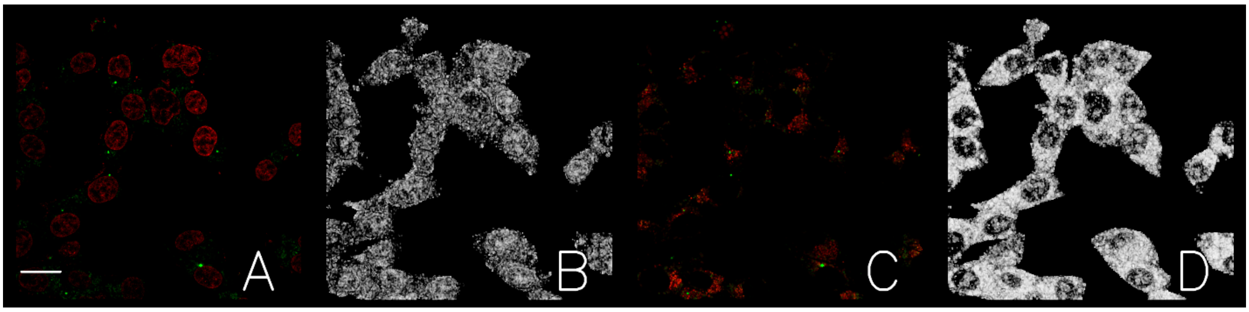

Figure S52. Costes' mask and spatial Pearson images were retrieved from **Figure S51.** with ImageJ using JACoP. [54,55] Costes' Mask: ImageJ JACoP performs Costes' automatic threshold analysis. The created mask highlights the co-localized regions of the compared channels. [54,55] Costes's Mask, blue and green channels compared from **Figure S51.** partD (A); Spatial Pearson map, blue and green channels compared from **Figure S51.** partD (B); Costes' Mask, red and green channels compared from **Figure S51.** partD (C); Spatial Pearson map, red and green channels compared from **Figure S51.** partD (D).

Table S18. Based on our previous studies, [52,53] we have also evaluated the co-localization using ImageJ JACoP. [54,55]

		**Red-Green**	**Blue-Green**
**Pearson Coefficient**[a]		0.593	0.018
**Manders Coefficients**[b]	**M1**	0.943	0.517
(original)	**M2**	0.975	0.561

[a] This is a linear equation that uses linear regression to calculate the relationship between the intensities of two images. 1 value stands for complete correlation, and -1 stands for complete negative correlation, while zero stands for no correlation at all

[b]Manders' overlap coefficient is based on Pearson but takes out the average intensities. The values range from 0 to 1. M1 and M2 are relative values between the sum intensity of the channel, whereas the other channel has a value above zero compared to the total sum intensity of the channel. [54,55]

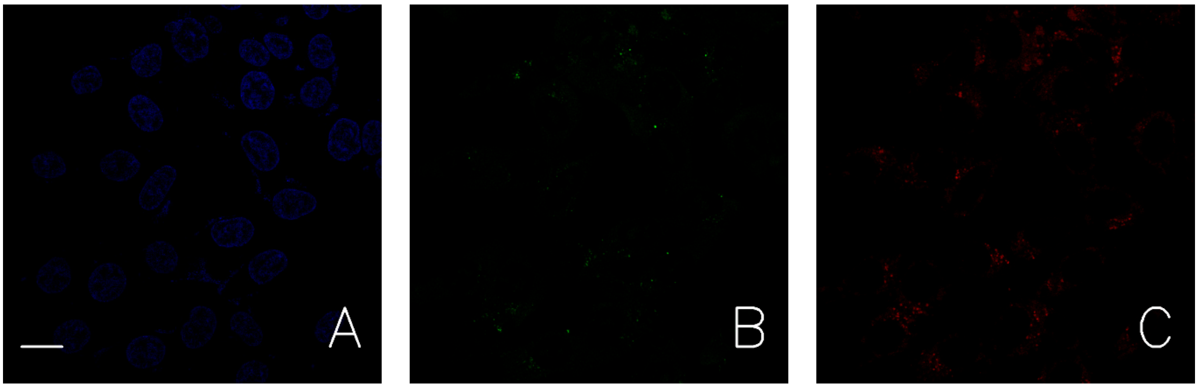

Figure S53. Internalization of peptide Cf-Arg$_6$-Lys(PCA) captured by confocal laser scanning microscopy using unprocessed images. Cells were incubated for 90 minutes with peptide (c=5 μM). Nuclei were stained with Hoechst 33342, blue (A); Cf labelled peptide, green (B); lysosomes were labelled with LysoTracker Deep Red, red (C). Cells were examined by Leica SP8 using an HC PL APO CS2 63x/1.40 OIL objective with the hybrid detector. The scale bar represents 20 μm.

Table S19. Co-localization profile of peptide Cf-Arg$_6$-Lys(PCA). We applied Leica LasX software using unprocessed images for image analysis and co-localization study. (A) red-green channels; (B) blue-green channels.

**(A)**	**Colocalization**	
Pearson's Correlation	0,4477	
Overlap Coefficient	0,5763	
Colocalization Rate	44,34%	
Colocalization Area [m²]	5,45E-11	
Area Image [m²]	2,10E-08	
Area Foreground [m²]	1,23E-10	
Area Background [m²]	2,09E-08	
	Channel Green	Channel Red
Mean Intensity Image	4,02	3,69
Mean Intensity Colocalization	33,63	54,49
Intensity Sum Image	4211436	3866252
Intensity Sum Colocalization	91643	148475
**(B)**	**Colocalization**	
Pearson's Correlation	0,1128	
Overlap Coefficient	0,3099	
Colocalization Rate	0,71%	
Colocalization Area [m²]	1,78E-12	
Area Image [m²]	2,10E-08	
Area Foreground [m²]	2,50E-10	
Area Background [m²]	2,07E-08	

	Channel Green	Channel Blue
Mean Intensity Image	7,11	4,02
Mean Intensity Colocalization	69,02	57,9
Intensity Sum Image	7455261	4211436
Intensity Sum Colocalization	6143	5153

Table S20. Co-localization analysis using single-channel confocal "tif" images (unprocessed images from Leica LasX software).

**Pearson Correlation** (Red-Green)	**Pearson Correlation** (Blue-Green)	**L_{coeff}** (Red)	**L_{coeff}** (Blue)	**Average Intensity** (Green in Red Uptake)	**Average Intensity** (Green in Blue Uptake)	**Average Intensity** (Green)
0.4427	0.1086	0.8838	0.8585	4.62	4.05	2.04

The „in-house Python-based" method calculates the "Pearson Correlations," the Lcoeff (Red, Blue), and the average intensity values of green channel intensities. We created categories by filtering the channels based on varying criteria. We also count the sum of the number of pixels of each category. We developed metrics based on channel overlap and used these metrics to calculate the L_{coeff} (Red, Blue).

[a]Value calculated by a Python module [50] from the flattened red and green channels. Relative value (-1-1) that reflects the linear correlation of two sets of the intensity values of the red and green channel pixels

[b]Value calculated by a Python module [50] from the flattened red and green channels. Relative value (-1-1) that reflects the linear correlation of two sets of the intensity values of the blue and green channel pixels

[c]Co-localization value calculated from red and green channel pixel counts employing the developed equation

[d]Co-localization value calculated from blue and green channel pixel counts employing the developed equation

[e]Calculated average intensity of peptides co-localized with lysosomes

[f]Calculated average intensity of peptides co-localized with nuclei

[g]Calculated average intensity of peptides in cell cytosol/cytoplasm

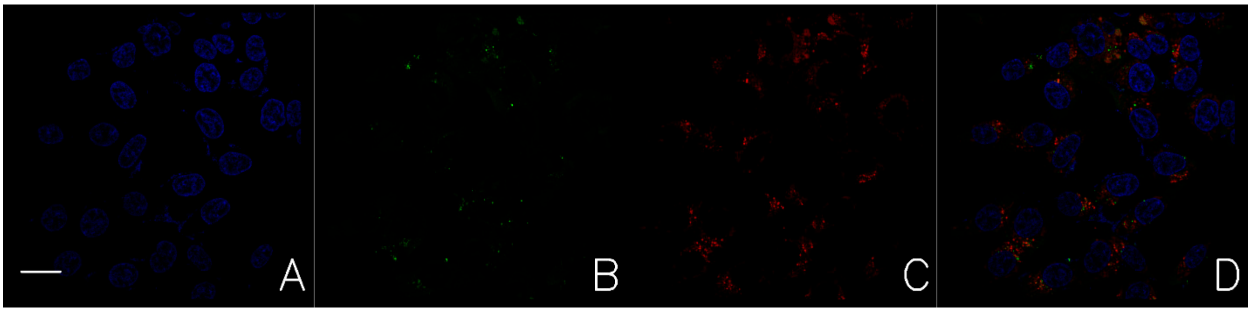

Figure S54. Internalization of peptide Cf-Arg$_6$-Lys(PCA) captured by confocal laser scanning microscopy using processed images. Cells were incubated for 90 minutes with peptide (c=5 μM). Nuclei were stained with Hoechst 33342, blue (A); Cf labelled peptide, green (B); lysosomes were labelled with LysoTracker Deep Red, red (C); merged channels (D). Cells were examined by Leica SP8 confocal microscope with adaptive lightning mode using an HC PL APO CS2 63x/1.40 OIL objective with the hybrid detector. The scale bar represents 20 μm.

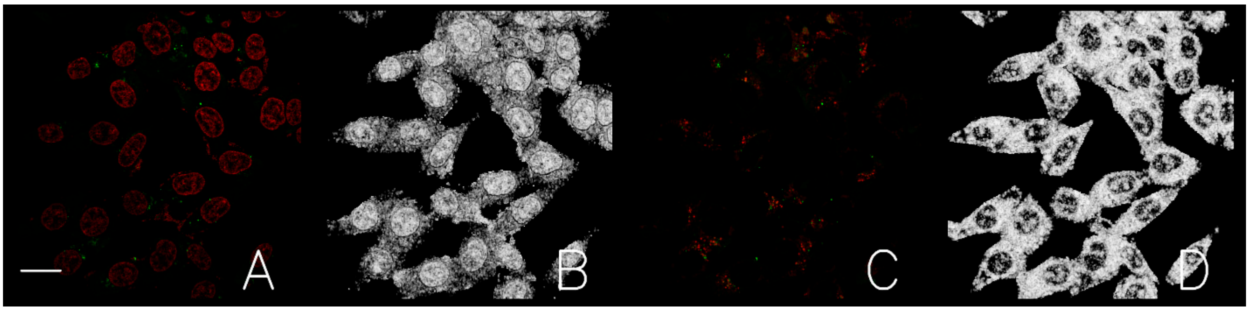

Figure S55. Costes' mask and spatial Pearson images were retrieved from **Figure S54.** with ImageJ using JACoP. [54,55] Costes' Mask: ImageJ JACoP performs Costes' automatic threshold analysis. The created mask highlights the co-localized regions of the compared channels. [54,55] Costes's Mask, blue and green channels compared from **Figure S54.** partD (A); Spatial Pearson map, blue and green channels compared from **Figure S54.** partD (B); Costes' Mask, red and green channels compared from **Figure S54.** partD (C); Spatial Pearson map, red and green channels compared from **Figure S54.** partD (D).

Table S21. Based on our previous studies, [52,53] we have also evaluated the co-localization using ImageJ JACoP. [54,55]

		Red-Green	Blue-Green
**Pearson Coefficient**[a]		0.487	0.062
**Manders Coefficients**[b]	**M1**	0.993	0.881
(original)	**M2**	0.819	0.596

[a] This is a linear equation that uses linear regression to calculate the relationship between the intensities of two images. 1 value stands for complete correlation, and -1 stands for complete negative correlation, while zero stands for no correlation at all

[b]Manders' overlap coefficient is based on Pearson but takes out the average intensities. The values range from 0 to 1. M1 and M2 are relative values between the sum intensity of the channel, whereas the other channel has a value above zero compared to the total sum intensity of the channel. [54,55]

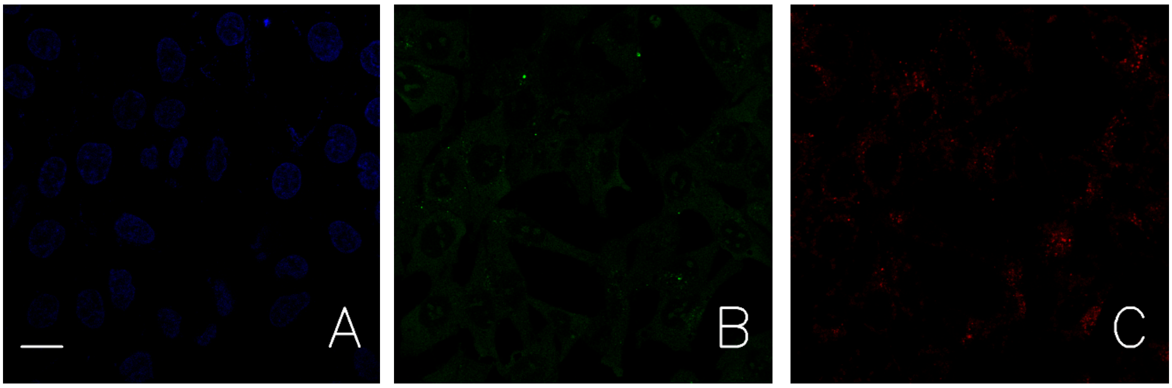

Figure S56. Internalization of peptide PCA-Arg$_6$-Lys(Cf) captured by confocal laser scanning microscopy using unprocessed images. Cells were incubated for 90 minutes with peptide (c=5 μM). Nuclei were stained with Hoechst 33342, blue (A); Cf labelled peptide, green (B); lysosomes were labelled with LysoTracker Deep Red, red (C). Cells were examined by Leica SP8 using an HC PL APO CS2 63x/1.40 OIL objective with the hybrid detector. The scale bar represents 20 μm.

Table S22. Co-localization profile of peptide PCA-Arg$_6$-Lys(Cf). We applied Leica LasX software using unprocessed images for image analysis and co-localization study. (A) red-green channels; (B) blue-green channels.

**(A)**	**Colocalization**	
Pearson's Correlation	0,4106	
Overlap Coefficient	0,6167	
Colocalization Rate	66,92%	
Colocalization Area [m^2]	1,32E-10	
Area Image [m^2]	2,10E-08	
Area Foreground [m^2]	1,98E-10	
Area Background [m^2]	2,08E-08	
	Channel Green	Channel Red
Mean Intensity Image	11,43	5,54
Mean Intensity Colocalization	34,83	57,16
Intensity Sum Image	1,20E+07	5813466
Intensity Sum Colocalization	230304	377963
**(B)**	**Colocalization**	
Pearson's Correlation	0,0947	
Overlap Coefficient	0,3612	
Colocalization Rate	8,53%	
Colocalization Area [m^2]	1,71E-11	
Area Image [m^2]	2,10E-08	
Area Foreground [m^2]	2,00E-10	
Area Background [m^2]	2,08E-08	

	Channel Green	Channel Blue
Mean Intensity Image	6,93	11,43
Mean Intensity Colocalization	80,95	36,35
Intensity Sum Image	7265196	1,20E+07
Intensity Sum Colocalization	69051	31004

Table S23. Co-localization analysis using single-channel confocal "tif" images (unprocessed images from Leica LasX software).

**Pearson Correlation** (Red-Green)[a]	**Pearson Correlation** (Blue-Green)[b]	**L_{coeff}** (Red)[c]	**L_{coeff}** (Blue)[d]	**Average Intensity** (Green in Red Uptake)[e]	**Average Intensity** (Green in Blue Uptake)[f]	**Average Intensity** (Green)[g]
**0.4073**	**0.0968**	**0.9797**	**0.9790**	**9.27**	**8.48**	**3.76**

The „in-house Python-based" method calculates the "Pearson Correlations," the Lcoeff (Red, Blue), and the average intensity values of green channel intensities. We created categories by filtering the channels based on varying criteria. We also count the sum of the number of pixels of each category. We developed metrics based on channel overlap and used these metrics to calculate the L_{coeff} (Red, Blue).

[a]Value calculated by a Python module [50] from the flattened red and green channels. Relative value (-1-1) that reflects the linear correlation of two sets of the intensity values of the red and green channel pixels

[b]Value calculated by a Python module [50] from the flattened red and green channels. Relative value (-1-1) that reflects the linear correlation of two sets of the intensity values of the blue and green channel pixels

[c]Co-localization value calculated from red and green channel pixel counts employing the developed equation

[d]Co-localization value calculated from blue and green channel pixel counts employing the developed equation

[e]Calculated average intensity of peptides co-localized with lysosomes

[f]Calculated average intensity of peptides co-localized with nuclei

[g]Calculated average intensity of peptides in cell cytosol/cytoplasm

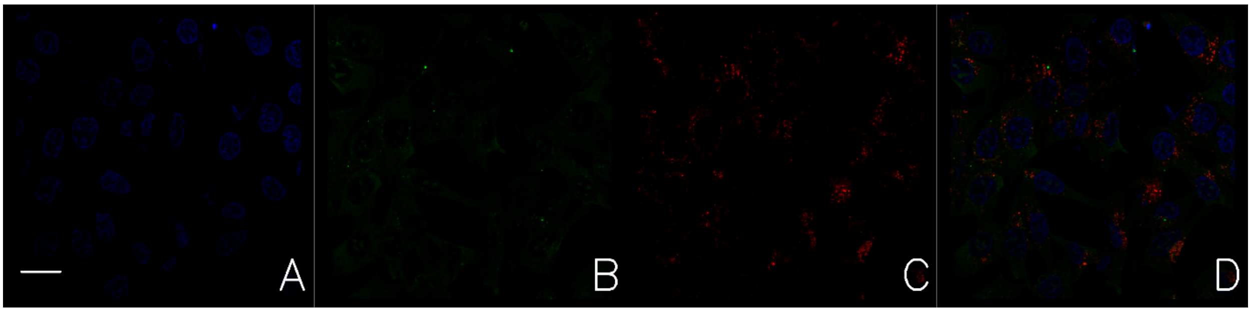

Figure S57. Internalization of peptide PCA-Arg$_6$-Lys(Cf) captured by confocal laser scanning microscopy using processed images. Cells were incubated for 90 minutes with peptide (c=5 μM). Nuclei were stained with Hoechst 33342, blue (A); Cf labelled peptide, green (B); lysosomes were labelled with LysoTracker Deep Red, red (C); merged channels (D). Cells were examined by Leica SP8 confocal microscope with adaptive lightning mode using an HC PL APO CS2 63x/1.40 OIL objective with the hybrid detector. The scale bar represents 20 μm.

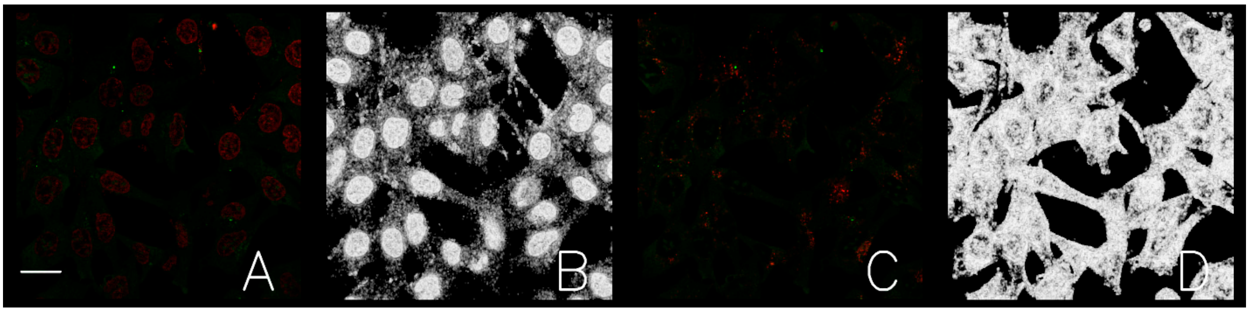

Figure S58. Costes' mask and spatial Pearson images were retrieved from **Figure S57.** with ImageJ using JACoP. [54,55] Costes' Mask: ImageJ JACoP performs Costes' automatic threshold analysis. The created mask highlights the co-localized regions of the compared channels. [54,55] Costes's Mask, blue and green channels compared from **Figure S57.** partD (A); Spatial Pearson map, blue and green channels compared from **Figure S57.** partD (B); Costes' Mask, red and green channels compared from **Figure S57.** partD (C); Spatial Pearson map, red and green channels compared from **Figure S57.** partD (D).

Table S24. Based on our previous studies, [52,53] we have also evaluated the co-localization using ImageJ JACoP. [54,55]

		Red-Green	Blue-Green
**Pearson Coefficient**[a]		0.434	0.149
**Manders Coefficients**[b]	**M1**	0.999	0.999
(original)	**M2**	0.818	0.484

[a] This is a linear equation that uses linear regression to calculate the relationship between the intensities of two images. 1 value stands for complete correlation, and -1 stands for complete negative correlation, while zero stands for no correlation at all

[b]Manders' overlap coefficient is based on Pearson but takes out the average intensities. The values range from 0 to 1. M1 and M2 are relative values between the sum intensity of the channel, whereas the other channel has a value above zero compared to the total sum intensity of the channel. [54,55].
